# Robust Regression Analysis of GCMS Data Reveals Differential Rewiring of Metabolic Networks in Hepatitis B and C Patients

**DOI:** 10.3390/metabo7040051

**Published:** 2017-10-08

**Authors:** Cedric Simillion, Nasser Semmo, Jeffrey R. Idle, Diren Beyoğlu

**Affiliations:** 1 Interfaculty Bioinformatics Unit and SIB Swiss Institute of Bioinformatics, University of Bern, Baltzerstrasse 6, 3012 Bern, Switzerland; cedric.simillion@dbmr.unibe.ch; 2 Department of BioMedical Research, University of Bern, Murtenstrasse 35, 3008 Bern, Switzerland; nasser.semmo@insel.ch (N.S.); jeffrey.idle@dbmr.unibe.ch (J.R.I.); 3 Department of Visceral Surgery and Medicine, Department of Hepatology, Inselspital, University Hospital of Bern, 3010 Bern, Switzerland; 4 Division of Systems Pharmacology and Pharmacogenomics, Samuel J. and Joan B. Williamson Institute, Arnold & Marie Schwartz College of Pharmacy and Health Sciences, Long Island University, Brooklyn, New York, NY 11201, USA

**Keywords:** metabolomics, robust regression analysis, metabolic perturbation networks, hepatitis B virus, hepatitis C virus, glycolysis, gluconeogenesis, pentose phosphate pathway, glucose transporters, TCA cycle

## Abstract

About one in 15 of the world’s population is chronically infected with either hepatitis virus B (HBV) or C (HCV), with enormous public health consequences. The metabolic alterations caused by these infections have never been directly compared and contrasted. We investigated groups of HBV-positive, HCV-positive, and uninfected healthy controls using gas chromatography-mass spectrometry analyses of their plasma and urine. A robust regression analysis of the metabolite data was conducted to reveal correlations between metabolite pairs. Ten metabolite correlations appeared for HBV plasma and urine, with 18 for HCV plasma and urine, none of which were present in the controls. Metabolic perturbation networks were constructed, which permitted a differential view of the HBV- and HCV-infected liver. HBV hepatitis was consistent with enhanced glucose uptake, glycolysis, and pentose phosphate pathway metabolism, the latter using xylitol and producing threonic acid, which may also be imported by glucose transporters. HCV hepatitis was consistent with impaired glucose uptake, glycolysis, and pentose phosphate pathway metabolism, with the tricarboxylic acid pathway fueled by branched-chain amino acids feeding gluconeogenesis and the hepatocellular loss of glucose, which most probably contributed to hyperglycemia. It is concluded that robust regression analyses can uncover metabolic rewiring in disease states.

## 1. Introduction

Hepatitis B virus (HBV) has been reported to infect some two billion persons, with greater than 350 million chronically infected [1]. In contrast, hepatitis C virus (HCV), which exists as seven variant genotypes [2], is believed to have infected 185 million persons [3]. The development of hepatocellular carcinoma (HCC) has a lifetime risk of 20% to 30% for untreated persons chronically infected with HBV, HCV, or both [4]. In an attempt to understand the mechanisms of infection and the progression of viral hepatitis to end-stage liver disease, both HBV [5,6] and HCV [5,7] attracted genomic and proteomic investigations around the new millennium. The investigation of the small molecule fingerprints of HBV and HCV infection using metabolomics lagged behind the genomic and proteomic efforts by almost a decade [8,9,10]. Metabolomics reveals information that is closer to the phenotype of the infected liver than do genomics, transcriptomics, or proteomics. While these other omics might predict, to certain degrees, the metabolic perturbations occurring post-infection with HBV or HCV, metabolomics can give a direct measurement of these, which can then be further augmented using transcriptomic data [3]. More detailed insights can be obtained in laboratory animals [11,12] or in cell culture [13,14] by analyzing isotopomers after the administration of [^13^C]glucose and/or [^13^C]glutamine, for example.

In human investigations, it is not possible to conduct some of these procedures. *Ex vivo* metabolomics on urine and plasma obtained from HBV- and HCV- infected persons must rely on a more static metabolite picture rather than the dynamic metabolism of isotopomers. Although several investigators have reported metabolomic data on HBV and HCV patients, investigations were in general conducted to discover biomarkers for HBV [10,15,16] and HCV [15,17,18,19] cirrhosis or fibrosis, together with HCC metabolism with HBV [1,15,20] and HCV [3,15,21] infection or for progression to HCC [22,23,24,25,26]. Studies have also simply investigated the impact of HBV [27,28,29,30] or HCV [8,27,31,32,33] infection on the metabolome or the metabolic consequences of drug treatment [2,34,35].

Many metabolomics reports, particularly the earlier ones, merely generate a list of up- and down-regulated metabolites in one condition versus another. It is important that metabolomic findings lead to mechanistic insights. For example, we described a metabolomic investigation of HCV infection using matched uninfected controls. The finding of downregulated urinary fructose and galactose combined with upregulated urinary sorbitol, galactitol, and xylitol led to the finding that the aldose reductase gene *AKR1B10* displayed a six-fold increased expression in HCV+ compared to HCV- liver biopsies [36]. This increased flux through the polyol pathway consumes NADPH, the obligatory cofactor in the generation of antioxidant GSH by glutathione reductase [37], and therefore is likely to contribute to elevated ROS and therefore to HCV hepatitis [36]. Gas chromatography-mass spectrometry (GCMS) is particularly adept at the detection and quantitation of small intermediary metabolites such as those comprising glycolysis [36], the TCA cycle [38], and the pentose phosphate pathway [39]. Robust bioinformatic procedures are required to identify the maximum number of metabolic networks that might be up- or down- regulated in particular biological circumstances, based upon urinary and plasma/serum GCMS metabolic profiles. In this report, we used regression analysis and revealed many changes in the correlations of metabolite pairs, indicating subtle differences between the metabolomes of HBV and HCV infected patients.

## 2. Results

### 2.1. Direct Comparison of Metabolite Intensities Shows Very Little Difference between HBV and HCV Patients

We expanded the previously reported GCMS dataset of 30 HCV patients with an additional 30 HBV patients. For each patient, we analyzed both a blood plasma and a urine sample. The same control plasma and urine donors were used for both datasets. Both datasets were merged and corrected for batch effects (see Materials and Methods). After excluding patients that had resolved the infection and outlier samples, our dataset contained data from 27 HBV patients, 19 HCV patients, and 26 healthy control subjects.

To account for the batch effect, we only included metabolites that were positively detected in both datasets, either in the control or patient samples, separately for each biofluid, namely, plasma or urine. In other words, metabolites detected only in one dataset were excluded from further analysis. It should be noted that, whenever a metabolite was observed in a given dataset, it was always detected in both the patient and control subjects. Thus, our analysis did not detect any metabolites that are unique to HBV or HCV infected patients. Table 1 lists the retained metabolites for both body fluids.

**Figure 1:**
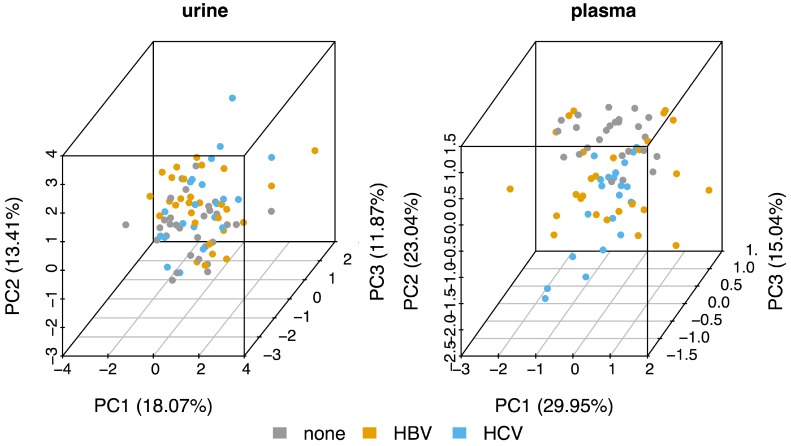
Principal component analysis (PCA) for the urine and plasma datasets.

As can be seen from the principal component analysis (PCA) plots in Figure 1, the metabolomic profiles of the urine samples (left panel) of the three sub-cohorts (control, HBV, and HCV) all cluster together. The control plasma samples (right panel) are somewhat separated from both the HBV and HCV samples, mainly along the second component (PC2). However, although the HBV samples seem to have a larger spread along PC1, the HBV and HCV plasma samples also separate quite poorly. Thus, the PCA suggests that there seems to be very little difference between the metabolomic profiles of HBV and HCV patients.

**Figure 2:**
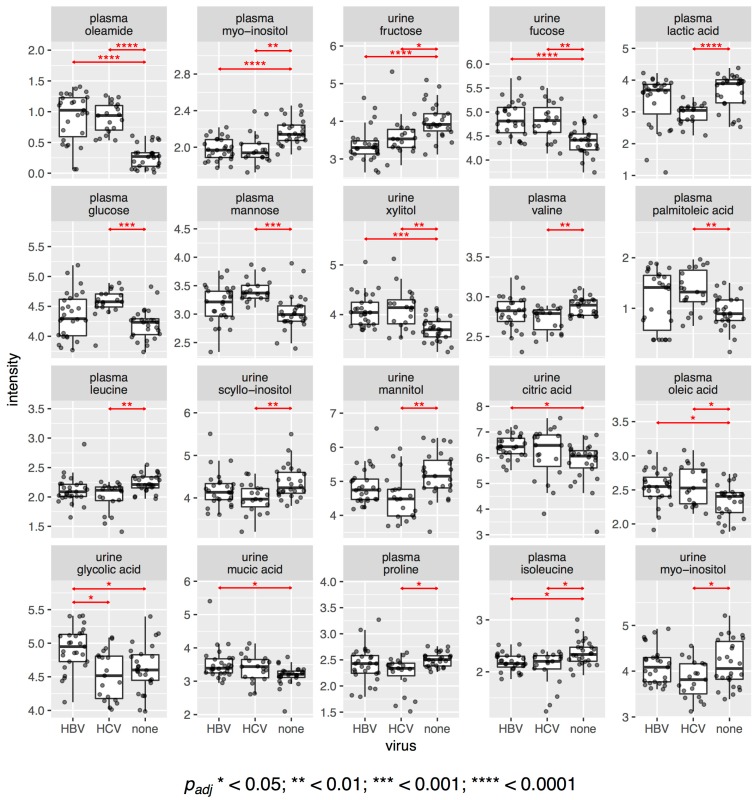
Dot and boxplots for the metabolites with statistically significantly different intensities between the three sub-cohorts. The *p* values are adjusted for multiple comparisons (Benjamini-Hochberg).

This observation is also confirmed when we look at the difference in intensities of individual metabolites. Figure 2 shows dot and boxplots for all metabolites that had statistically significantly different intensities between any of the three sub-cohorts. In total, we found 20 significant metabolites with an adjusted p value of 0.05 or less. Of those, 10 were different between HBV patients and the control samples and 17 were different between HCV patients and controls. Only one compound, urinary glycolic acid, was found to be statistically significantly different between HBV and HCV patients, with an adjusted p value of 0.022. It was also greater in HBV (+7%) and lesser in HCV (−2%) than in the controls. Two metabolites, urine citric acid (+8%) and urine mucic acid (+6%), only had higher intensities in HBV compared to the controls, whereas three metabolites, plasma glucose (+10%), plasma mannose (+13%), and plasma palmitoleic acid (+44%), only had higher intensities in HCV compared to the controls. Four metabolites were found to be elevated in both HBV and HCV: plasma oleamide (+300%, +260%, respectively), urine fucose (+8%, +8%, respectively), plasma oleic acid (+6%, +5%, respectively), and urine xylitol (+11%, +14%, respectively). No metabolite was found to have lower intensities in HBV only, but seven metabolites were diminished in HCV compared to the controls: plasma lactic acid (−23%), plasma valine (−3%), plasma leucine (−4%), plasma proline (−6%), urine *scyllo*-inositol (−5%), urine *myo*-inositol (−7%), and urine mannitol (−13%). Finally, three metabolites had lower levels in both HBV and HCV compared to the controls: plasma *myo*-inositol (−9%, −10%, respectively), plasma isoleucine (−7%, −4%, respectively), and urine fructose (−15%, −10%, respectively). The respective *p* values are given in Figure 2. We had previously reported some of these differences between HCV and the controls in a separate study [36]. In summary, HBV plasma differed statistically significantly from the control plasma, mainly due to an increase in the lipids oleamide and oleic acid. HCV plasma, similarly, differed from the control plasma with increased lipids (oleamide, palmitoleic acid, and oleic acid); increased sugars, namely, glucose and mannose; decreased lactic acid; and decreased amino acids, namely, proline and the essential branched-chain amino acids (BCAAs), valine, leucine, and isoleucine. HBV and HCV urinary metabolite patterns were more similar to those for plasma (Figure 2).

### 2.2. Regression Analysis Reveals Different Metabolite Correlation Patterns between HBV and HCV Patients

**Figure 3:**
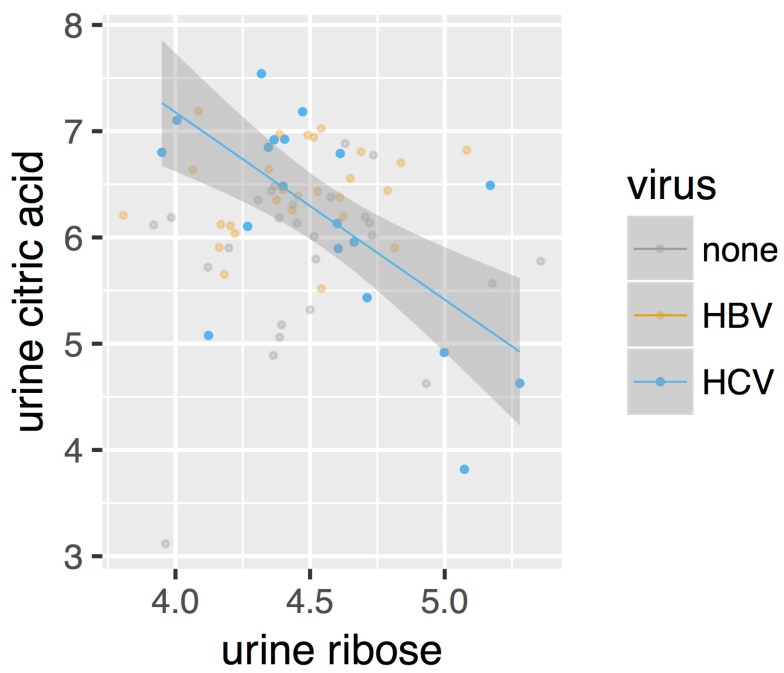
Scatterplot of the intensities of urine ribose and urine citric acid. Only the correlation in HCV (regression line and 95% confidence interval shown) is statistically significant.

After directly comparing the metabolite intensities, we next constructed metabolic perturbation networks for both HBV and HCV. These networks are based on the observation that, for some metabolite pairs, the correlation of their intensities changes between the different sub-cohorts in our dataset. Figure 3 gives such an example. As can be seen from this figure, no correlation is observed for the control cohorts (marked as ‘none’ in the legend), nor for the HBV patients. A negative correlation, however, can be observed for the HCV cohort. This observation suggests that a metabolomic process involving both of these compounds is at play in HCV patients, which is otherwise absent in control individuals as well as in HBV patients.

To investigate to the global extent of this phenomenon, we used robust linear regression models to detect all pairs of metabolites where the correlation pattern changes between the control cohort and either the HBV or HCV patients (see Materials and Methods). We then summarized all these cases into a disease-specific metabolic perturbation network for each virus. In these networks, the nodes represent metabolites and the edges represent the correlation patterns between them. We discriminate between four different types of edges. The first type is called ‘appear’ and refers to cases in which no correlation is observed in the control group but one appears in the virus group. Likewise, the ‘disappear’ type represents cases in which an observation is present in the control group but disappears in the virus group. Additionally, we also define the ‘flip’ type, which occurs when a positive correlation is observed in one group and a negative one in the other. Finally, we also consider ‘change’ relations, which denote the cases in which the slope of the regression line changes significantly between both groups, without affecting the sign—positive or negative—of the correlation. See the Materials and Methods section for a description of how these types are determined statistically.

**Figure 4:**
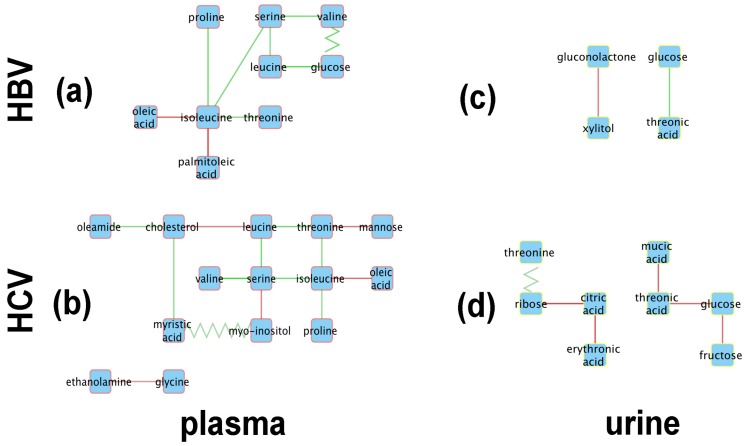
Metabolic perturbation networks for (**a**,**c**) HBV and (**b**,**d**) HCV in plasma (**a**,**b)** (red node borders) and urine (**c**,**d**) (yellow node borders). Solid lines denote ‘appear’ type edges and zigzag lines ‘flip’ edges. The edge color reflects the slope of the correlation in HBV/HCV patients, with green denoting positive and red negative correlations.

Figure 4 shows the resulting metabolic perturbation networks for HBV and HCV in plasma and urine. Surprisingly, the vast majority of edges are of the ‘appear’ type, as listed in Table 2. For both networks together, 28 ‘appear’ edges were found, compared to only three ‘flip’ edges, and no ‘change’ or ‘disappear’ edges. Another striking observation is that, for both HBV and HCV, the metabolic perturbation networks are much bigger in plasma compared to urine. Indeed, taken together over both virus types, we detected 23 perturbation edges in plasma compared to eight in urine, which is a statistically significant difference ($\chi^{2}$-test; p=0.0071).

When totaling the number of perturbations across virus types, it can be seen that the HCV network is bigger, with 20 edges compared to only 11 edges in the HBV network. Although this difference is not statistically significant ($\chi^{2}$-test; p=0.11), it is still remarkable, given that we included only 19 HCV patients in our analysis, compared to 27 HBV patients, which would actually reduce the statistical power to detect correlations in the HCV cohort.

From a qualitative point of view, it is striking that the metabolic perturbation networks of HBV and HCV are fairly similar in plasma, sharing seven nodes, whereas the urine networks only have two metabolites in common (Figure 4). Indeed, the HBV and HCV plasma networks have a common ‘backbone’ of an edge between serine and isoleucine, as well as most of the edges connected to the latter two nodes. The only unique features of the HBV plasma metabolic perturbation network are the ‘flip’ edge between valine and glucose and the ‘appear’ edge between isoleucine and palmitoleic acid. Interestingly, the nodes unique to the HCV network are related to the lipid metabolism, apart from mannose and glycine. Ethanolamine and *myo*-inositol are head-groups of phospholipids; myristic acid and oleamide are, respectively, a saturated and a monounsaturated fatty acid; and cholesterol is a key lipid. The urine networks only share two nodes, glucose and threonic acid. Although both nodes are connected by an ‘appear’ edge in both networks, the sign of the correlation is opposite between HBV and HCV.

### 2.3. Mechanistic Interpretations of the Metabolic Perturbation Networks

#### 2.3.1. General Considerations

It should be recalled that the PCA plots for both the plasma and urine datasets showed clustering of the HBV, HCV, and control data, suggesting little, if any, difference in the plasma and urine metabolomes for any of the three datasets (Figure 1). However, of the 39 metabolites (Table 1) included in the analysis, 20 showed statistically significant differences between the HBV, HCV, and control datasets. Furthermore, the robust regression analysis, involving 490 regressions, 190 for the plasma metabolites and 300 for the urinary metabolites, produced the metabolic perturbation networks in Figure 4. 

**Figure A1:**
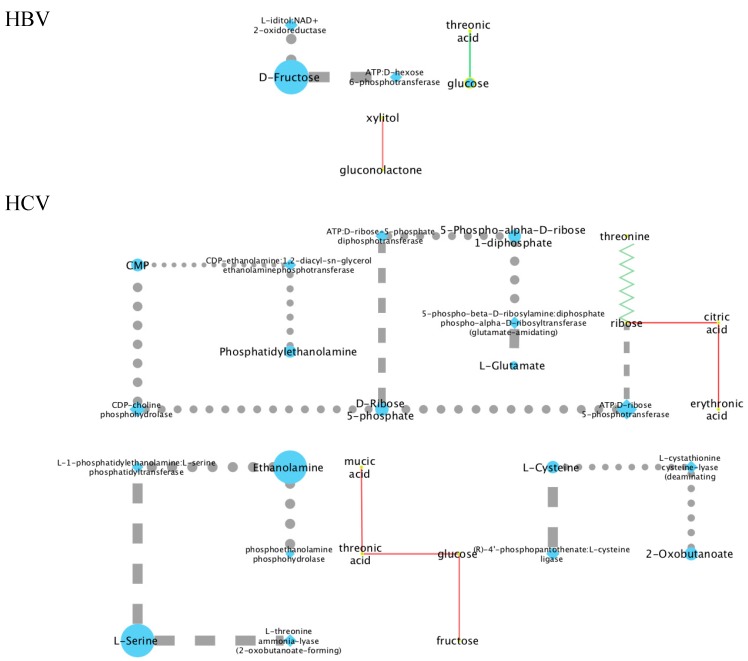
Integrated perturbation and metabolic network for the HBV and HCV urine samples. Circle nodes represent metabolites, and diamonds represent enzymes. Dashed lines represent enzyme-substrate relations, and dotted lines enzyme-product relations; solid lines are appearing correlations in the metabolic perturbation network (Figure 4). The size of the node and thickness of the edge represent the relative significance.

**Figure A2:**
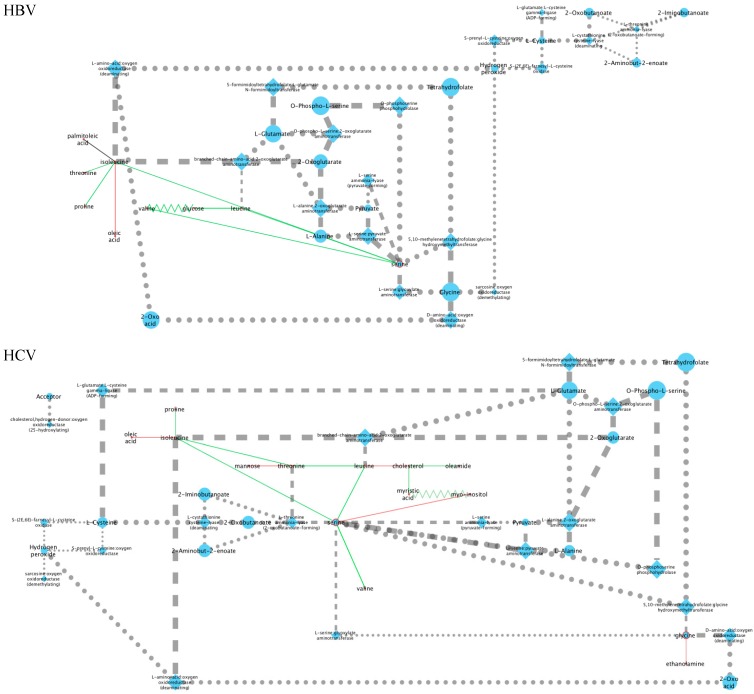
Integrated perturbation and metabolic network for the HBV and HCV plasma samples. Circle nodes represent metabolites, and diamonds represent enzymes. The same legend applies as in Figure A1.

The first approach to understanding the binary metabolite relationships described by the perturbation networks was to create a metabolic network by retrieving all human enzyme-substrate and enzyme-product relations from the KEGG database. For each pair of metabolites connected by an edge in a metabolic perturbation network, we then detected all the shortest paths between these in the metabolic network. We then compared the number of times each node or edge occurs in these shortest paths to the same number in randomized networks (see Materials and Methods) to obtain, for each metabolic perturbation network, a statistically significant metabolic network (Appendix A). Figure A1 and Figure A2 show these metabolic networks for urine and plasma, respectively. It was found that this approach resulted in a complex outcome that was difficult to interpret. It was therefore decided to proceed using the published literature to interpret the positive and negative correlations in plasma and in urine for both HBV and HCV.

#### 2.3.2. Analysis of the HBV and HCV Metabolic Perturbation Networks

As can be seen in Figure 4a, the central node of the HBV plasma network is isoleucine, which is connected to oleic acid, palmitoleic acid, threonine, proline, and serine, the last of which is a node connected to valine and leucine and then to glucose. Interestingly, the edges connecting isoleucine to other amino acids (threonine, serine, proline) all consist of positive correlations, while the connections to the two monounsaturated fatty acids (MUFAs), oleic acid and palmitoleic acid, represent negative correlations. The oleic acid-isoleucine negative correlation (Figure 4a) is consistent with the observations that plasma oleic acid was greater in HBV than in the controls and that plasma isoleucine was lower in HBV than in the controls (Figure 2). 

The HBV urine network is comprised of just two correlations, a negative correlation between gluconolactone and xylitol and a positive correlation between glucose and threonic acid (Figure 4c). All four of these cellular metabolites have relationships to the pentose phosphate pathway (PPP). Glucose and xylitol feed the PPP through glucose-6-phosphate and xylulose/xylulose-5-phosphate, respectively [42]. Gluconolactone and threonic acid are metabolites produced by the PPP from glucose and xylitol, respectively [42]. 

The HCV metabolic perturbation networks contain more correlations than their HBV counterparts (Figure 4b,d). The central node in the HCV plasma network (Figure 4b) is serine, which is connected directly to the three BCAAs, leucine, isoleucine, and valine (positive correlations), and also to *myo*-inositol (negative correlation). Leucine is negatively correlated with valine but positively correlated with threonine. Other negative correlations in HCV plasma worthy of note are between valine and cholesterol, isoleucine with oleic acid (common to HBV plasma; Figure 4a), and glycine with ethanolamine. In this last case, serine is decarboxylated to ethanolamine, leading in two further steps to glycolic acid, whereas glycine is transaminated to glyoxylic acid, which is reduced to glycolic acid. The negative correlation between glycine and ethanolamine would be explained by fluxes in these pathways, especially as glycine and serine are metabolically interchangeable [43]. 

The urinary ribose and citrate negative correlation (Figure 4d) may be a direct effect of the HCV virus. It has been reported that monocyte derived macrophages infected with HIV-1 or HIV-2 displayed elevated ribose 5-phosphate, a PPP intermediate, with no change in TCA cycle intermediates such as citrate when compared with noninfected cells [44]. Citric acid was also negatively correlated with L-erythronic acid ((2*R*,3*R*)-2,3,4-trihydroxybutanoic acid), the diastereomer of threonic acid [45], in the HCV urinary network (Figure 4d). In summary:

- The initial PCA analysis of plasma and urine datasets suggested that there were few differences between the HBV+, HCV+, and control metabolomes.
- Univariate statistics gave 28 statistically significant differences for a subset of 20 metabolites; 15 in plasma and 13 in urine.
- Robust regression analysis revealed networks of correlations between pairs of metabolites that mainly appear in HBV+ and HCV+ plasma and urine but are not observed in healthy controls.
- The positive and negative correlations provided novel insights into the HBV+ liver compared with the HCV+ liver.

## 3. Discussion

Multiple metabolomic studies on HBV+ and HCV+ patients have been reported. For HBV, the studies mostly sought biomarkers of the development of cirrhosis or HCC [24,46,47,48,49] or biomarkers simply of HBV infection itself [10,28]. Similar studies have been reported in the case of HCV for serum biomarkers of progression to HCC [21,22,23] and for hepatic fibrosis [17,19]. Since HCV infection can be treated, several reports deal with metabolomic changes before and after treatment that were interpreted as reflecting the metabolic effects of the virus [2,34]. Finally, urine analysis by NMR has been proposed as a means of early detection of HCV infection [8]. 

In contrast to the aforementioned studies, ours was the first to compare HBV+ with HCV+ patients, together with uninfected healthy controls. Furthermore, both plasma and urine were analyzed. Our aim was to understand the differential footprints of HBV and HCV on the liver by uncovering the covariation between metabolites. Therefore, a direct comparison between the two viral infections was made using the same control group of healthy subjects. It was not our objective to discover biomarkers for HBV and HCV infection, as the literature is replete with such data. Our intention was to tease out statistically significant correlations, both positive and negative, between pairs of metabolites to create what we have called ‘metabolic perturbation networks’. To generate metabolic perturbation networks, we conducted a robust regression analysis for both plasma and urinary metabolites, involving 490 regressions. ‘Perturbation’ refers to the fact that some correlations appeared in, say, HBV or HCV patients that were not present in the controls, and a few flipped their correlation from positive to negative between HBV and HCV patients. Using this analysis, it was believed that metabolic differences between the HBV-infected and the HCV-infected liver could be identified.

It is pertinent to discuss whether or not the differences between HBV and HCV patients, together with the metabolite correlations we observed, could be due to spurious factors. Firstly, bloods were collected from all patients, according to a standardized protocol in the hepatology clinic, and the plasma was quickly prepared and frozen at -80°C. Furthermore, all patients were fasted for a minimum of 6 h. The samples were analyzed by GCMS using the same laboratory procedure for all batches, run on the same instrument and by the same analyst. However, the HBV patients were statistically significantly younger than the HCV patients, with significantly lower plasma ALT values. These observations with age and ALT were not unexpected, for they have been reported by others in a large European study [50]. Moreover, a positive smoking history and current use of medication were statistically significantly more common in HCV patients than in HBV patients. However, the occurrence of diabetes, fasting blood glucose levels, and BMI values did not differ statistically between the two patient groups. Therefore, we do not believe that dietary factors or host metabolic factors contributed significantly to the metabolic findings that are ascribed here to chronic HBV or HCV infection. Furthermore, the stage of hepatic fibrosis as determined by the METAVIR score was not statistically significantly different between HBV (2.1 ± 1.4) and HCV (3.1 ± 0.9) patients and therefore did not appear to be related to the metabolic findings presented here.

Amino acids are a source of cellular energy by the anaplerotic feeding of the TCA cycle via intermediates such as pyruvate, 2-oxoglutarate, and succinyl-CoA [51], which may explain the multiple correlations in plasma amino acid concentrations reported for fasted patients [52]. Moreover, HBV appears to elevate amino acid levels in concert in hepatocytes [29], perhaps explaining the positive amino acid correlations described here for HBV plasma (Figure 4a). Finally, branched-chain amino acids (BCAAs), particularly leucine but also isoleucine and valine, regulate protein translation and, therefore, amino acid levels, involving mTORC1 activation [53] by the HBV HBx protein [54]. In addition, HBx has been shown to activate fatty acid oxidation [55], fueling the TCA cycle with increased acetyl-CoA and thus will decrease the demand on amino acid anaplerosis. This may explain the negative correlations of HBV plasma for isoleucine with both oleic acid and palmitoleic acid.

Regarding xylitol in the urine of HBV patients, this polyol was found to exhibit the largest decline of any urinary metabolite detected between control samples and liver cirrhosis/hepatocellular carcinoma patients in Egypt [56]. Furthermore, it was reported over four decades ago that patients infused with xylitol showed grossly increased urinary excretion of threonic acid, interpreted as arising through the PPP [42,43]. In addition, L-threonic acid ((2*R*,3*S*)-2,3,4-trihydroxybutanoic acid) is also an oxidative metabolite of ascorbic acid formed via dehydroascorbic acid (DHA) [57,58] that is believed to be transported into cells by glucose transporters that also transport DHA [59]. Therefore, the cotransport of glucose and threonic acid may also explain the positive correlation between these two metabolites in HBV urine or, in addition, the conversion of glucose through the PPP to yield threonic acid [42,43]. It has also recently been suggested that elevated threonic acid, as an oxidative metabolite of ascorbic acid, is a measure of an attenuated oxidative stress response [60].

As stated above, the only metabolite that was statistically significantly different between HBV and HCV was urinary glycolic acid (Figure 2), reflecting differential glycine and serine metabolism between these two virus infections. Concerning the leucine and valine negative correlation, mild liver damage caused by the administration of 2 g CCl_4_ to rats resulted in a rise in plasma valine and a fall in plasma leucine 16 h post-administration. With 4 g and 8 g doses, more severe liver damage was recorded, together with rises in most plasma amino acids [61]. HCV hepatitis may mimic these amino acid changes. Regarding the valine and cholesterol negative correlation in HCV plasma, metabolic syndrome was reported to be distinguished from obesity by increased valine, leucine, and isoleucine degradation [62]. Both conditions are associated with elevated serum cholesterol. Furthermore, rats fed a diet supplemented with BCAAs and had no change in serum total cholesterol but displayed a lower hepatic cholesterol deposition [63]. Finally, since cholesterol and BCAAs are potential energy metabolites, it is reasonable that their plasma levels might be negatively correlated. Both serine and *myo*-inositol are components of relatively minor membrane phospholipids. *Myo*-inositol is incorporated directly into phosphatidylinositol in the endoplasmic reticulum (ER). Phosphatidylserine is synthesized from phosphatidylcholine or phosphatidylethanolamine, also in the ER [64]. However, there is competition between serine and *myo*-inositol for the pathways that lead to these phospholipids [64,65], perhaps explaining their negative correlation in HCV plasma. We have previously reported that plasma mannose is elevated in HCV+ plasma [36], and this is reflected in Figure 2. Mannose was correlated only with threonine in HCV plasma. Protein mannosylation is a common glycosylation pattern for HCV [66], and threonine protein residues are a principal site of mannosylation [67]. However, it has not been reported that free threonine can be mannosylated or if the amino acid can inhibit protein mannosylation, despite the fact that *O*-α-D-mannopyranosyl-L-threonine is available from several commercial sources. This would have furnished an explanation for the negative correlation between mannose and threonine. Finally, isoleucine and oleic acid were negatively correlated in HCV (and also HBV) plasma. Oleic acid was higher in both HBV and HCV plasma than in control plasma, while isoleucine was lower in both HBV and HCV plasma than in control plasma (Figure 2). Interestingly, a rat model for postoperative fatigue syndrome, involving both 30% and 70% partial hepatectomies, displayed a progressive declining trend from control to 30% hepatectomy to 70% hepatectomy for a number of serum metabolites, including isoleucine. Concomitantly, there was an increasing trend across the same samples for several other metabolites, including oleic acid [68]. One possible explanation for the decline in plasma isoleucine, a BCAA, is that it is used as an energy source, as has been reported for fatigued human subjects [69]. Under such circumstances, increased fatty acid mobilization may occur as a potential energy source, explaining the rise in plasma oleic acid [68]. This would also suggest that both HCV+ and HBV+ patients behave metabolically like patients with postoperative fatigue syndrome. 

Regarding the positive correlation between citric acid and erythronic acid in HCV urine (Figure 4d), the literature gives little guidance on this, except that both were uncorrelated in the plasma of patients with acute ischemic stroke [70] and that urinary erythronic acid was massively elevated in the transaldolase deficiency of the PPP. In this ischemic stroke study, citric acid was not measured, but two additional TCA metabolites, 2-oxoglutarate and fumarate, were also massively increased in urine [45]. Finally, erythronic acid was one of three upregulated metabolites in both plasma and urine in response to a single oral dose of either 50 or 100 g sucrose [71]. It is believed that erythronic acid is increased when there is diminished flux through the PPP, as in the case of transaldolase deficiency [45]. Erythronic acid has therefore been proposed to be generated by an as yet unknown alternative pathway to the PPP [45,71]. This as yet undescribed pathway may be related to the TCA cycle since erythronic acid was negatively correlated with citric acid in HCV urine (Figure 4d). While the PPP appeared active in HBV liver, the data suggest that it may be impaired in HCV liver. 

The negative correlation between urinary mucic acid and threonic acid in HCV is also of interest (Figure 4d). Mucic acid is the dicarboxylic acid of galactose and can be fermented by several species of the intestinal microbiota such as *Escherichia coli* and *Salmonella enteritidis* [72]. Threonic acid is elevated in the serum of mice with experimental intestinal ischemia [73], which may have been the result of altered gut floral metabolism. This negative correlation in HCV may result from alterations in the intestinal microbiota metabolism. As indicated above, we report a positive correlation between threonic acid and glucose in HBV urine (Figure 4c), but, in HCV urine, a negative correlation was found for these two metabolites (Figure 4), a clear distinguishing metabolic feature between HBV and HCV hepatitis. This observation in HCV is a similar finding to the changes found in intestinal ischemia in mice [73]. HCV+ patients tend to be hyperglycemic, as shown in Figure 2 and as has been discussed elsewhere [36]. Glucose transport may underlie the relationship between glucose and threonic acid. The question, therefore, is whether or not glucose transporters operate differently in HBV+ and HCV+ livers. In the case of HBV, the deletion mutant pre-S2 protein is capable of activating the mTOR signal cascade [74], which leads to the translocation of the SLC2A1 (GLUT1) transporter protein to the hepatocyte membrane, enhanced cellular glucose uptake, and aerobic glycolysis (‘Warburg effect’) [75]. By contrast, the HCV NS5A protein promotes hepatic gluconeogenesis [76] through phosphoenolpyruvate carboxykinase (PEPCK), which is in part regulated by the energy metabolism of the TCA cycle [77,78]. NS5A also suppresses the membrane expression of the SLC2A1 and SLC2A2 (GLUT2) transporters and therefore glucose uptake into liver [76,79]. These mechanisms are believed to contribute to the hyperglycemia frequently associated with HCV infection, at least for HCV genotype 3 [80]. Moreover, the polyol pathway is upregulated by HCV, whereby glucose is reduced to sorbitol by aldose reductase AKR1B10, which is then oxidized by sorbitol dehydrogenase to fructose [36]. NADPH is exchanged for NADH in this pathway, which compromises NADPH-dependent reactions such as glutathione reductase and nitric oxide synthase [37]. These differential mechanisms of glucose homeostasis may underpin the divergent correlations of glucose with threonic acid and glucose with fructose in HBV and HCV urines. 

This analysis is consistent with the HBV+ liver importing glucose via the SLC2A1 and SLC2A2 transporters and activating glycolysis and PPP but attenuating the TCA cycle. In comparison, this analysis is consistent with the HCV+ liver not importing glucose but generating it by gluconeogenesis. The analysis is also consistent with threonic acid being imported by the HBV+ liver using glucose transporters and, in addition, producing by PPP as a result of xylitol metabolism. Additionally, the analysis is consistent with glycolysis and PPP being downregulated in the HCV+ liver, with a consequent decrease in plasma lactic acid, and is also consistent with intracellular glucose leaking from the cell and contributing to the hyperglycemia frequently observed with chronic HCV infection. Finally, the analysis is consistent with the BCAAs valine, leucine, and isoleucine being metabolized in the HCV+ liver via the TCA cycle, thus lowering their plasma concentrations and bolstering gluconeogenesis.

Our bioinformatic investigation of the GCMS data resulting from the analysis of plasma and urine samples from HBV+ and HCV+ patients, together with HBV- and HCV- controls, has produced divergent metabolic pictures of the HBV and HCV infected liver. In the case of HBV infection, it could be interpreted that glucose was actively imported and used to fuel glycolysis and the PPP. In the case of HCV infection, the liver appeared not to import glucose but rather to synthesize it by gluconeogenesis using TCA cycle intermediates in part derived from BCAAs. We derive these interpretations using the following published observations. Our plasma analyses revealed that all three BCAAs were statistically significantly depressed in HCV+ patients. We have interpreted this as enhanced anaplerosis, which helps to fuel gluconeogenesis. After several catabolic steps, both leucine and isoleucine enter the TCA cycle as acetyl-CoA, and valine enters via succinyl-CoA. The expression of all the genes that regulate the rate-limiting steps of BCAA catabolism (*BCAT2*, *DBT*, *DLD*, *BCKDHA*, and *BCKDHB*) is correlated with insulin sensitivity [81]. HCV infection has long been recognized as being associated with type 2 diabetes mellitus (T2DM) [82], and a mouse model expressing the full HCV ORF has recently established that impaired glucose metabolism is a probable cause [83]. Of note was that this mouse model had reduced the hepatocyte membrane expression of SLC2A2 and displayed an attenuated uptake of glucose and higher hepatic glucose production. These data are consistent with our metabolomic findings, described above. The combination of reduced glucose uptake and impaired insulin signaling in these HCV protein expressing mice was interpreted as the cause of insulin resistance [83]. Given the effects of BCAA catabolism on insulin sensitivity [81], what role might BCAAs play in reversing insulin resistance? Several clinical investigators have administered BCAAs to HCV infected patients in attempts to improve prognosis [84]. This area is controversial, with some commentators suggesting that BCAAs can activate mTORC1 and cause insulin resistance [85], despite the observed beneficial effects to HCV infected patients of BCAA supplements or a BCAA-rich diet [84,86]. These relationships between BCAAs, gluconeogenesis, and hyperglycemia in HCV patients are implicit in our metabolomic model, described herein.

In general, metabolomic investigations using case-control protocols for diseases such as viral hepatitis have produced lists of latent biomarkers that have then been subjected to receiver operating characteristic (ROC) curve analysis to evaluate their suitability as classifiers (for example, the progression of hepatitis [24] or cirrhosis [46] to HCC or distinguishing between different grades of HCC [33]). Furthermore, metabolite set enrichment analysis has been reported in metabolomic studies of this kind, which use, for example, the MetaboAnalyst 3.0 online algorithm (http://www.metaboanalyst.ca) [87,88]. Groups of discovered metabolites are often placed schematically into boxes labeled, for example, phospholipid metabolism, long-chain fatty acids, or steroid biosynthesis [87]. In addition, some studies have undertaken network analysis, the stringing together of the observed list of elevated metabolites in a disease state into an annotated network that contains cognate biochemical pathways [16,24]. However, these are merely graphical representations of a metabolite list, enhanced or attenuated in a particular state. 

We have taken a different approach. Plasma and urine metabolites are not always the products of metabolic pathways or their substrates. They may be elevated or reduced in these biofluids as a result of alterations in membrane transporters due or in some other way related to the disease state. Moreover, a metabolite may not be acting merely as an enzyme substrate; it may inhibit another pathway competitively or noncompetitively. In addition, non-peptide neurotransmitters may be discovered by metabolomics in plasma or urine in various disease states, and, therefore, the appearance of molecules such as acetylcholine or norepinephrine would most likely to be due to neuronal activity rather than direct biosynthesis. The approach described here, that is, using robust regression analysis to generate metabolic perturbation networks, sought to uncover correlations, both positive and negative, between pairs of metabolites in both HBV and HCV infected persons. Rather than a list, these metabolic perturbation networks point clearly to the differential metabolic rewiring in HBV and HCV infection, as reported here. 

## 4. Materials and Methods 

### 4.1. Patient Selection

We created a combined HBV and HCV patient cohort by complementing the HCV cohort from our previous study [36] with a new HBV cohort. In both cases, the patients were positive for the respective virus but had not developed liver cirrhosis (LC) or HCC. In total, our combined cohort consisted of 30 HBV patients and 30 HCV patients chosen from the Bern Hepatology Biobank. All gave their written informed consent to donate blood and urine to the biobank, and the study was conducted according to the World Medical Association Declaration of Helsinki. The clinical investigation of HBV and HCV patient material was approved by the Ethics Commission of the Canton of Bern (Die Ethikkommission des Kantons Bern; reference number KEK 141/14). The HBV group consisted of 16 women and 14 men; the HCV group consisted of 15 women and 15 men. The patient age in the HBV group ranged from 20 to 66 years, with a mean (±S.D.) age of 40.6 ± 10.5 years, and, in the HCV group, it ranged from 28 to 73 years, with a mean of 50.0 ± 11.1 years (*p* < 0.01). For the HBV and HCV patients, the average BMIs (Kg/m^2^) were 26.1 ± 5.2 and 25.0 ± 5.2 (n.s.), respectively. For the HBV and HCV patients, the fasting blood glucose levels were 5.3 ± 1.4 and 5.0 ± 0.7 (n.s.), respectively, the plasma alanine aminotransferase levels (ALT; IU/l) were 49.9 ± 58.2 and 87.2 ± 62.1 (*p* < 0.01), respectively, and the METAVIR staging (degree of fibrosis) was 2.1 ± 1.4 and 3.1 ± 0.9 (n.s.), respectively. Diabetes had been diagnosed for three of 30 patients in both groups. A smoking history was determined for seven of 30 HBV and 23 of 30 HCV patients (χ^2^ = 17.1; *p* < 0.0001), and medication was taken by seven of 30 HBV and 20 of 30 HCV patients (χ^2^ = 11.4; *p* < 0.001). For this study, we excluded one HBV and six HCV patients that had resolved the infection, either spontaneously or after antiviral therapy.

For each patient, both a urine and a blood plasma sample was taken from the biobank for metabolomic profiling. The blood had been collected into EDTA tubes after a minimum of a 6 h fast, and, according to strict protocol in the hepatology clinic of the Inselspital Teaching Hospital, the plasma was prepared within 30 min and stored at −80 °C. Additionally, we used the same collection of control samples that were previously described [36]. This collection consisted of 30 urine samples from healthy volunteers (15 F, 15 M; aged 18 to 63 years), who were staff in the university and hospital departments, as well as 30 plasma samples from the local blood bank, from voluntary blood donors from all over Switzerland (15 F, 15 M; aged 41 to 55 years). All samples tested negative for the presence of antibodies and/or antigens for HIV, HCV, and HBV. All samples were also negative for syphilis. HIV, HBV, and HCV PCR testing was also negative for all control samples.

### 4.2. Gas Chromatography-Mass Spectrometry (GCMS) Analysis of Plasma and Urine

For the HBV, HCV, and control cohorts, both plasma and urine were profiled on the same GCMS instrument by the same operator after double derivatization and using 4-chlorophenylacetic acid as internal standard, as described [36,39,73,89]. Annotation of the gas chromatographic peaks was made upon both comparisons of the mass spectra with the NIST 14 library spectra collection of 276, 248 mass spectra and comparisons of the retention times and mass spectra of an in-house collection of 120 authentic compounds, as described previously [39,89].

### 4.3. Data Processing, Batch Correction, and Integration

For each patient cohort, HBV and HCV, and each sample type, urine and plasma, a separate dataset was created, consisting of patient and control samples. In each dataset, the relative intensities were processed as follows:

1. A matrix I was constructed in which the columns represent the samples and the rows the measured metabolites
2. A floor value, f, was calculated as $f=\lfloor log_{2}\left(I_{i,j}\right)\rfloor$ for all elements $I_{i,j}$ of I, where $I_{i,j}>0$
3. All elements of I, where $I_{i,j}=0$, are assigned a value of $2^{f}$
4. The entire matrix is now transformed such that $I^{\prime }=log_{2}\left(I\right)-f$
5. A vector, m, is calculated as the column means of $I^{\prime }$, as well as a vector, s, as the column standard deviations. A vector, o, is defined as o=m−min(m)
6. The vector, o, is subtracted from each row of $I^{\prime }$
7. Each row of $I^{\prime }$ is divided by s
8. Finally, all values of $I^{\prime }$ are offset by a value such that the lowest value of I is zero.
9. The result of these operations is a matrix in which all columns have the same mean and standard deviation.

The replicate samples—columns in the matrix $I^{\prime }$—are averaged in order to obtain a final intensity matrix, J, with as many columns as subjects, both patients and controls, in the cohort.

For each sample type, the matrices $J_{HBV}$ and $J_{HCV}$ are merged by first identifying the set of compounds C that were identified in both datasets. A merged matrix, M, is then created by selecting from $J_{HBV}$ and $J_{HCV}$ those rows that correspond to the compounds in C and by concatenating the columns of both sub-matrices. The resulting matrix is then batch-corrected using the method described by Leek et al. [90]. This step results in two single matrices, $M_{plasma}$, containing data for 20 metabolites, and $M_{urine}$, containing 25 metabolites.

Finally, $M_{plasma}$ and $M_{urine}$ are merged into one big matrix, $M_{merged}$, by concatenating their rows. This matrix is then used for all subsequent analyses and will from here on simply be referred to as M.

### 4.4. Detection of Differentially Abundant Metabolites

Metabolites that have statistically significant different abundances between two sample groups were detected using an unpaired t test. The resulting p-values were corrected for multiple testing using the Benjamini-Hochberg method.

### 4.5. Robust Regression Analysis

For each pair of metabolites X,Y, i.e., each pair of rows in the matrix M, five different robust linear models are fitted, each corresponding to a different scenario about how the intensity of Y, denoted as y, correlates to the intensity of X, denoted as x, as well as the patient group g:

1. A null model, with a single intercept, representing the case that y is independent of both x and g.
2. An intercept-only model, with a different intercept per subject group, modelling the case that y only varies with g.
3. A single intercept, single slope model, for the case in which y only correlates with x and is independent of g
4. A multiple intercept, single slope model, representing the case in which y depends on both x and g but in which the slope does not vary between the different patient groups.
5. An interaction model, where y depends on both x and g, as well as the interaction between them, meaning that the slope changes between the patient groups.

For both the multiple intercept and the interaction models, the reference for both the intercept and the interaction terms is the control cohort. As a result, the interaction terms reflect the difference between the regression slope coefficients of the HBV and HCV patients and the control subjects. The models were fitted using the rlm function from the MASS package in R [91]. Akaike’s information criterion (AIC) [27] was then used to determine which of these models fit the data best. Each time the interaction model had the best fit, a Wald test was used to calculate the significance of the interaction terms of this model. In all other cases, the p-value was assumed to be 1. The p-values for all models were then corrected for multiple testing using the Benjamini-Hochberg method.

All models with an adjusted p-value of 0.01 or less were retained. For each model, 95% confidence intervals were constructed for the general slope coefficient and the interaction terms for both HBV and HCV. Whenever this interval included zero, the corresponding coefficient was set to zero. Next, for each virus type, a network was constructed in which the nodes represent metabolites and the edges the models describing the correlation between them. Depending on the values of the general slope coefficient and the interaction coefficients for the respective virus, the edges were assigned to one of these types:

- When the interaction coefficient for the virus was zero, the edge was not included in the network.
- When the general slope was zero but the interaction coefficient was different from zero, the edge type was called ‘appear’
- When the general slope was different from zero but the interaction coefficient was zero, the edge type was called ‘disappear’
- When the both the general slope and the interaction coefficient were different from zero and the slope of the HBV or HCV patient group had a different sign than the general slope, the edge type was called ‘flip’. Note that the slope of a patient group is calculated as the sum of the general slope and the interaction term.
- When both the general slope and the interaction term were different from zero, the edge type was called ‘change’.

The resulting networks are referred to in the main text as metabolic perturbation networks.

### 4.6. Integration with Metabolic Networks

The collection of known human enzyme-substrate and enzyme-product annotations was retrieved from the KEGG database using the KEGGREST R module. For enzymes with unspecific substrate or product annotations, such as ‘a carboxylate’, specific annotations, e.g., 4-hydroxyphenylacetate, were added manually. These annotations were then merged into a bi-partite graph in which the nodes represented either enzymes or compounds and the edges represented substrate-enzyme or product-enzyme relations.

This graph was then used as an undirected metabolic network to find possible metabolic pathways between pairs of compounds found to be connected in a metabolic perturbation network. More precisely, for every edge E connecting the metabolites X and Y in a metabolic perturbation network, all possible shortest paths between X and Y in the metabolic network were retrieved, if any. The output of this step is an integrated network consisting of the original metabolic perturbation network and the metabolic shortest paths.

To assess whether the detected shortest metabolic paths are specific to the metabolic perturbation networks, we observed artefacts caused by the presence of hub nodes in the metabolic network, and the following Monte Carlo approach was adopted. For each node and metabolic edge in the integrated network, an inclusion score indicating the number of times it was included in any of the metabolic shortest paths, excluding start and end nodes, is recorded. Next, 1000 Monte Carlo replicates of the original metabolic perturbation network are created by first randomly selecting n nodes from the entire set of metabolites detected in our dataset and then creating the e edges between the selected nodes, where n and e are, respectively, the number of nodes and edges in the original metabolic perturbation network. These random networks were then integrated with the metabolic network in the same way as the original network, and the inclusion scores for these nodes and edges were also determined. This step allows us to compare the inclusion score of a given node or edge in the original integrated network to its distribution of scores in the randomized networks. Applying a confidence interval of 0.95, we consider an inclusion score in the original network to be significant if there, at most, 50 random networks with the same score or a higher score for that particular node or edge.

## Figures and Tables

**Table 1:** List of metabolites included in the merged dataset.

Metabolite	RT (min)	Plasma	Urine
lactic acid	13.32	X	X
glycolic acid	13.80	-	X
*p*-cresol	16.36	-	X
valine	19.28	X	-
urea	20.20	X	-
ethanolamine	21.05	X	-
leucine	21.24	X	-
isoleucine	21.98	X	-
proline	22.08	X	-
glycine	22.42	X	X
serine	24.30	X	X
threonine	25.21	X	X
threitol	28.38	-	X
erythronic acid	29.73	-	X
threonic acid	30.79	-	X
ribose *	30.97 33.29 33.71	-	X
4-hydroxyphenylacetic acid	32.10	-	X
xylitol	34.56	-	X
arabitol	34.90	-	X
fucose	35.06	-	X
citric acid	37.95	-	X
HPHPA **	37.96	-	X
myristic acid	38.11	X	-
gluconolactone	39.15	-	X
fructose	39.55	-	X
glucose ***	39.92 40.25	X	X
mannose	40.69	X	-
tyrosine	40.79	X	-
mannitol	41.02	-	X
gluconic acid	42.20	-	X
palmitoleic acid	42.46	X	-
mucic acid	42.54	-	X
*scyllo*-inositol	42.76	-	X
*myo*-inositol	44.78	X	X
oleic acid	46.80	X	-
stearic acid	47.33	X	-
oleamide	50.53	X	-
sucrose	53.72	-	X
cholesterol	61.47	X	-

* Ribose runs as three peaks due to an unmethoxymated silyl derivative, together with the (*E*)- and (*Z*)-isomers of the *O*-methyloxime silyl derivative of the ribose aldehyde. ** 3-(3-Hydroxyphenyl)-3-hydroxypropionic acid, a gut microbiota metabolite produced by *Clostridia* spp. from phenylalanine and related to autism and schizophrenia, as are 4-hydroxyphenylacetic acid and *p*-cresol, also observed in hepatitis B virus (HBV) and hepatitis C virus (HCV) patient urine (see above) [40,41]. *** Glucose runs as two peaks due to the (*E*)- and (*Z*)-isomers of the *O*-methyloxime silyl derivative produced by derivatization of the glucose aldehyde. RT means retention time (min).

**Table 2:** Counts of the different edge types for the metabolic perturbation networks of each virus in each fluid.

		Appear	Flip
HBV	plasma	8	1
	urine	2	0
HCV	plasma	13	1
	urine	5	1

## Supplementary Materials

The following are available online at www.mdpi.com/2218-1989/7/4/51/s1, Figure S1: plasma isoleucine and plasma serine, Figure S2: plasma isoleucine and plasma threonine, Figure S3: plasma leucine and plasma glucose, Figure S4: plasma leucine and plasma serine, Figure S5: plasma myristic acid and plasma myo−inositol, Figure S6: urine ribose and urine citric acid, Figure S7: urine threonine and urine ribose, Figure S8: urine threonic acid and urine mucic acid, Figure S9: urine erythronic acid and urine citric acid, Figure S10: plasma myristic acid and plasma cholesterol, Figure S11: plasma valine and plasma serine, Figure S12: plasma isoleucine and plasma palmitoleic acid, Figure S13: plasma isoleucine and plasma oleic acid, Figure S14: plasma leucine and plasma threonine, Figure S15: plasma isoleucine and plasma proline, Figure S16: urine xylitol and urine gluconolactone, Figure S17: plasma valine and plasma glucose, Figure S18: urine threonic acid and urine glucose, Figure S19: urine fructose and urine glucose, Figure S20: plasma ethanolamine and plasma glycine, Figure S21: plasma threonine and plasma mannose, Figure S22: plasma serine and plasma myo−inositol, Figure S23: plasma oleamide and plasma cholesterol, Figure S24: plasma leucine and plasma cholesterol.
