# Supplementary material for: Robust Regression Analysis of GCMS Data Reveals Differential Rewiring of Metabolic Networks in Hepatitis B and C Patients
Source: Metabolites. 2017 Oct 8;7(4):51. doi: 10.3390/metabo7040051 (PMC5746731; doi:10.3390/metabo7040051)

# Supplementary Figures

# Robust Regression Analysis of GCMS Data Reveals Differential Rewiring of Metabolic Networks in Hepatitis B and C Patients

**Cedric Simillion [1,2], Nasser Semmo [2,3], Jeffrey R. Idle [2,3], and Diren Beyoğlu [2,*]**

[1] Interfaculty Bioinformatics Unit and SIB Swiss Institute of Bioinformatics, University of Bern, Baltzerstrasse 6, 3012 Bern, Switzerland; cedric.simillion@dkf.unibe.ch

[2] Department of BioMedical Research, University of Bern, Murtenstrasse 35, 3008 Bern, Switzerland; cedric.simillion@dkf.unibe.ch; jeffrey.idle@dkf.unibe.ch; diren.beyoglu@dkf.unibe.ch

[3] Department of Visceral Surgery and Medicine, Department of Hepatology, Inselspital, University Hospital of Bern, 3010 Bern, Switzerland; nasser.semmo@insel.ch

Figure S1. plasma isoleucine and plasma serine

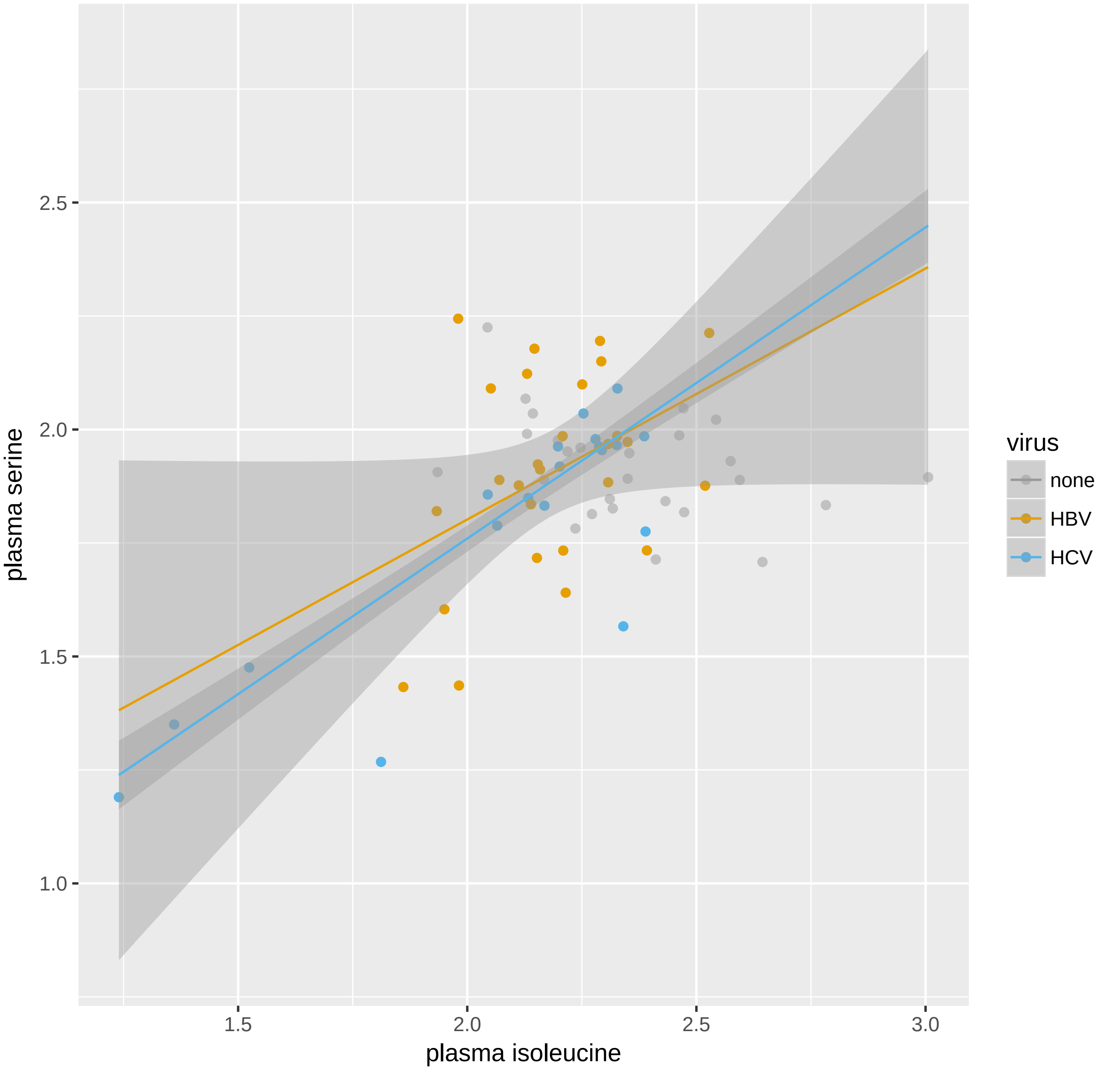

Figure S2. plasma isoleucine and plasma threonine

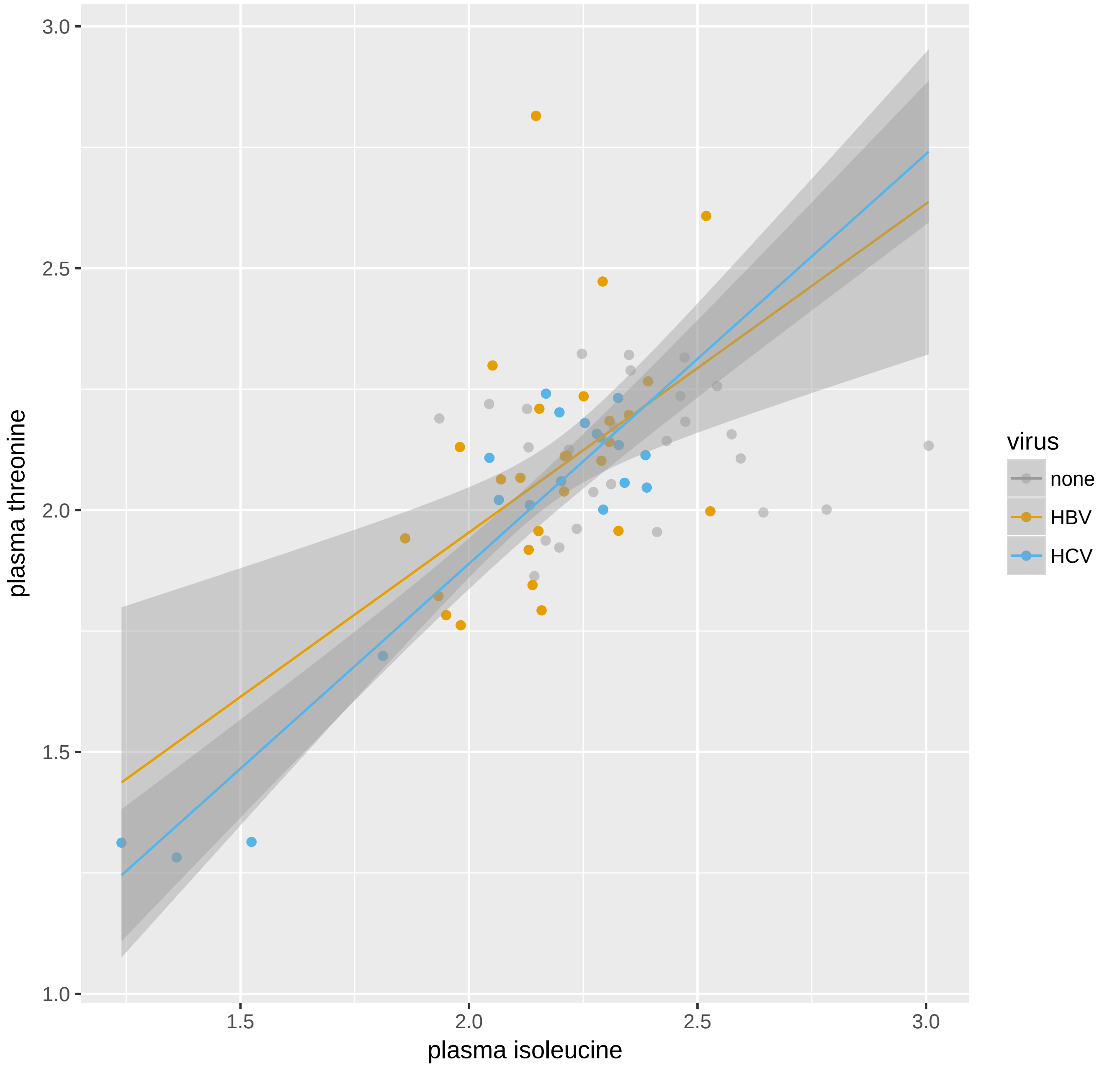

# Figure S3. plasma leucine and plasma glucose

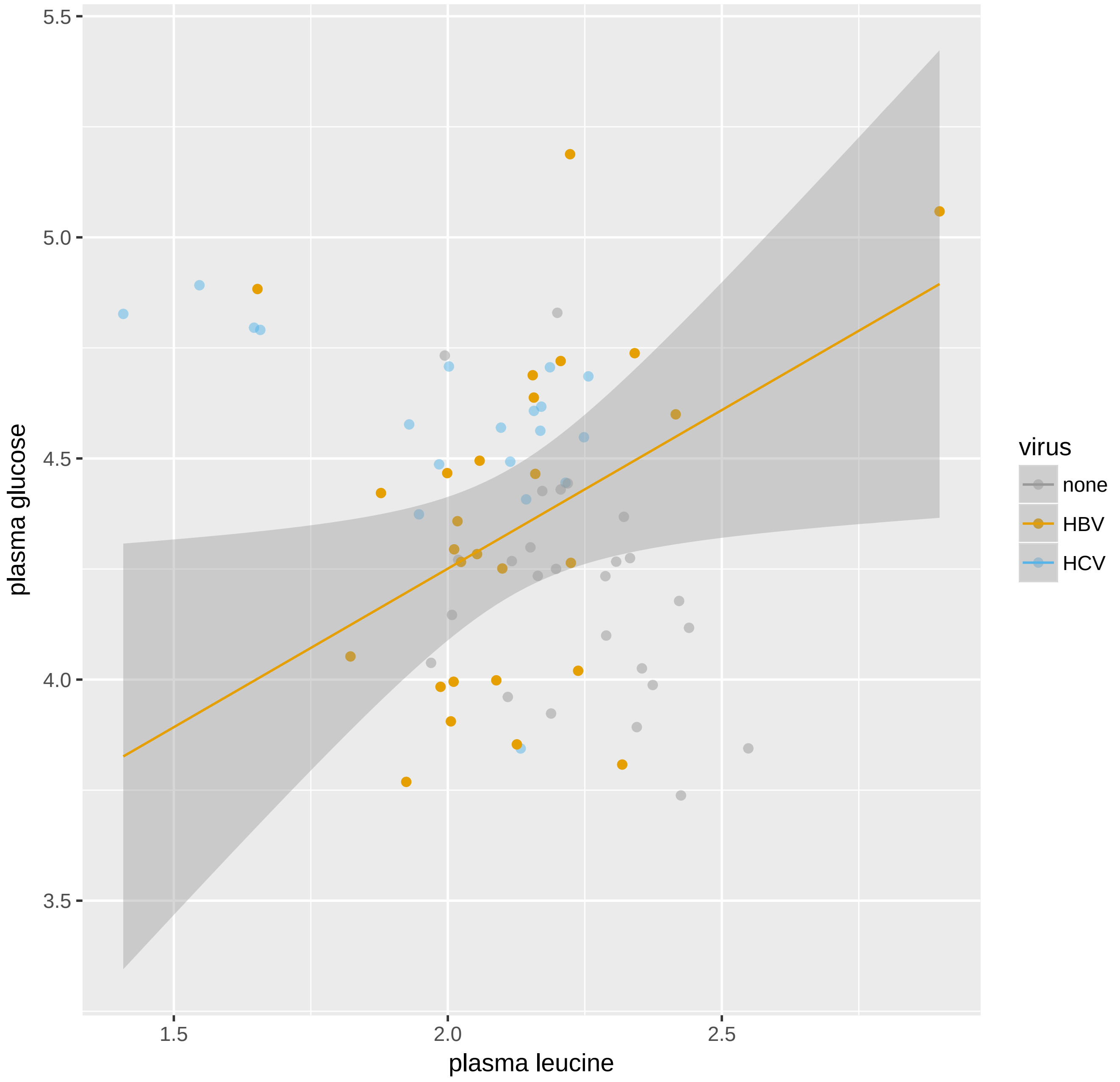

Figure S4. plasma leucine and plasma serine

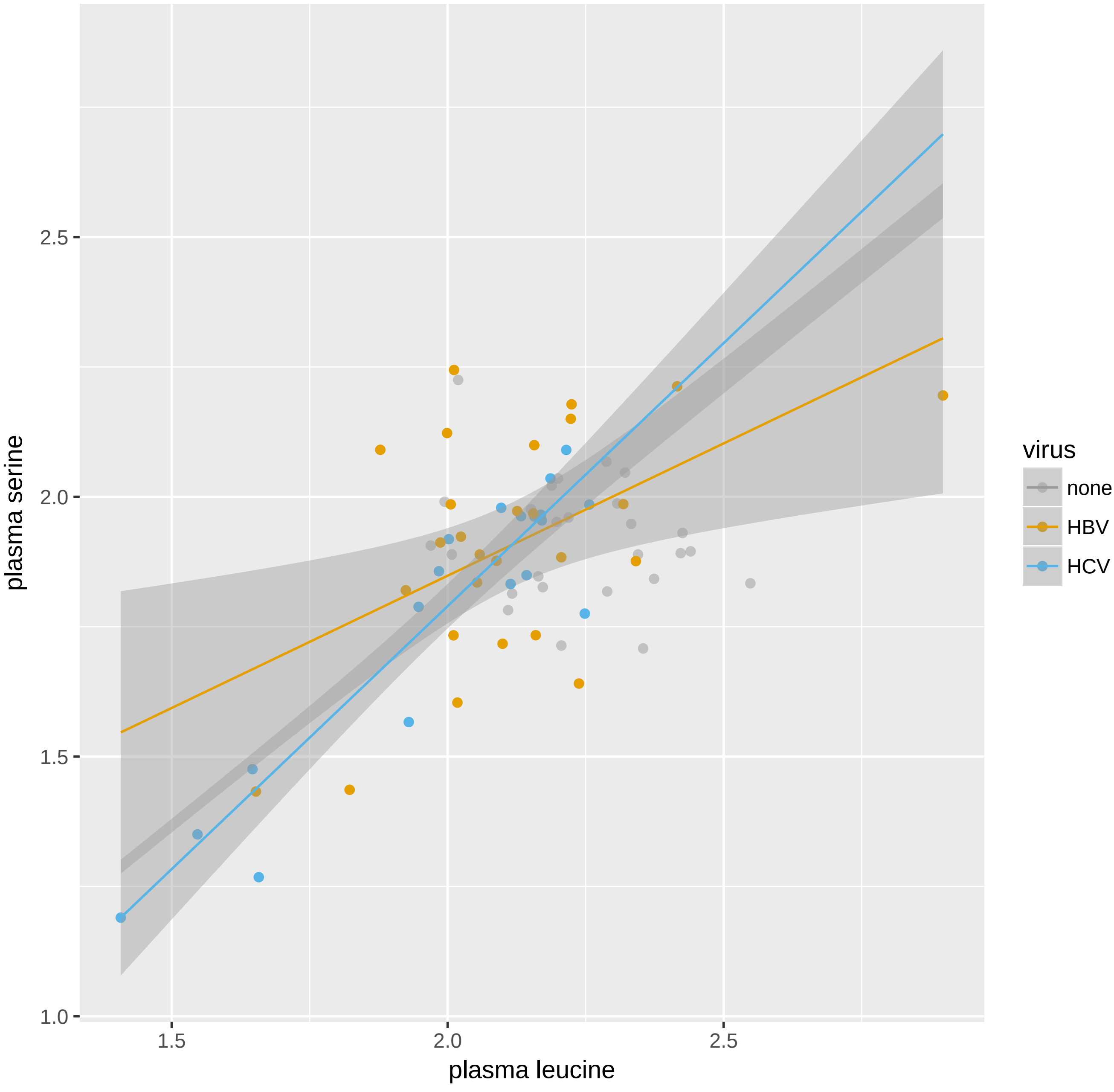

Figure S5. plasma myristic acid and plasma myo−inositol

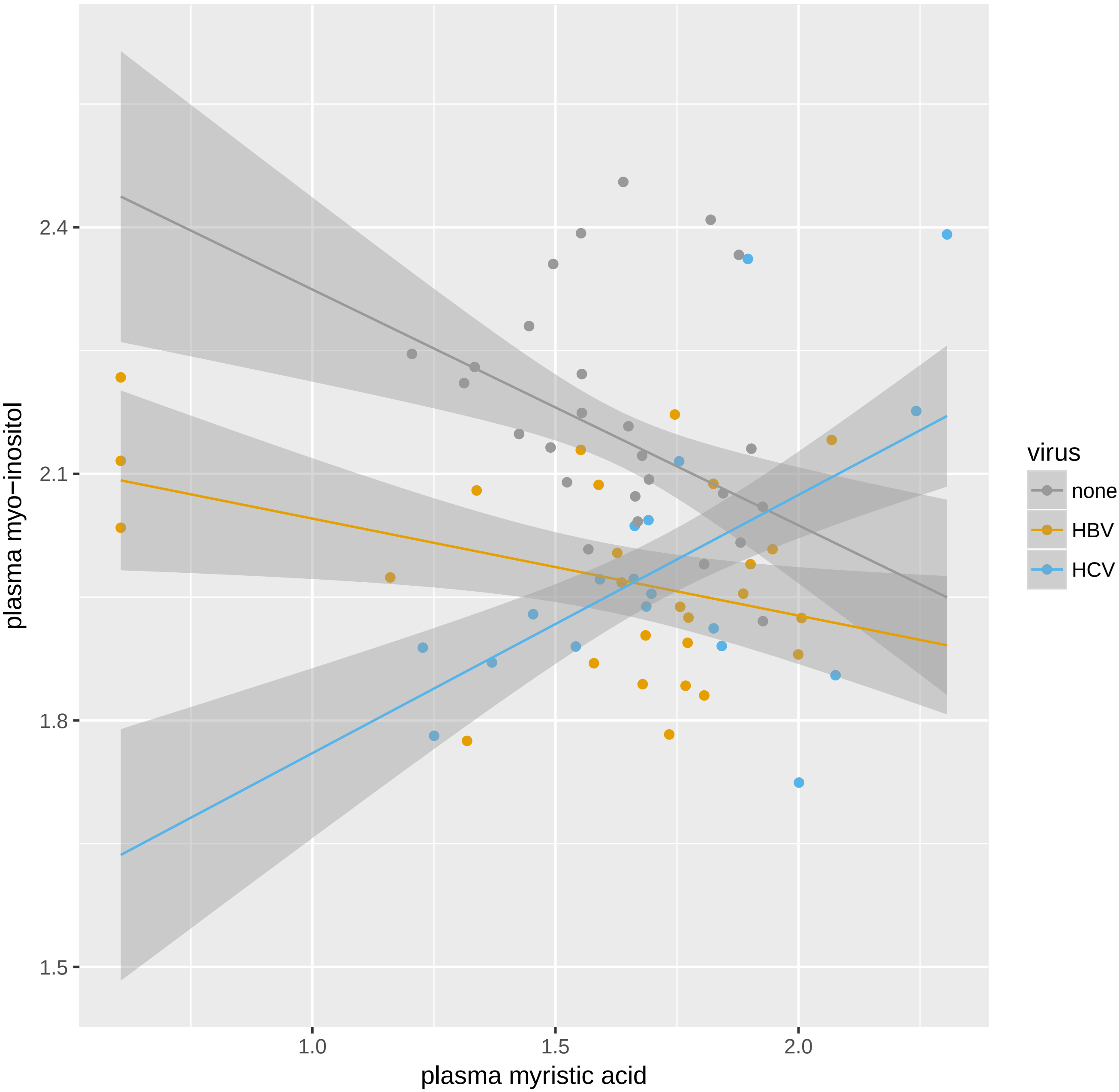

Figure S6. urine ribose and urine citric acid

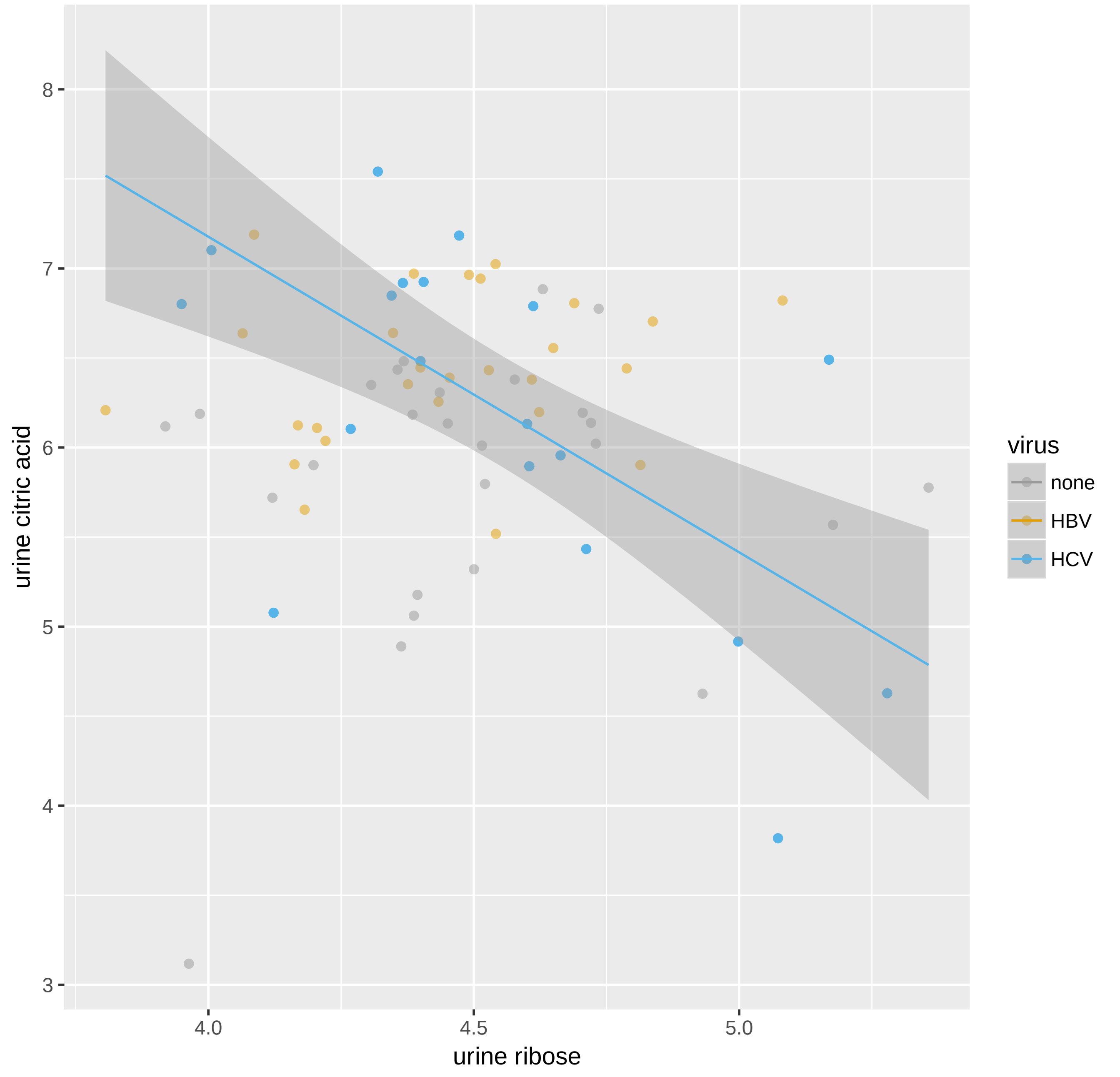

# Figure S7. urine threonine and urine ribose

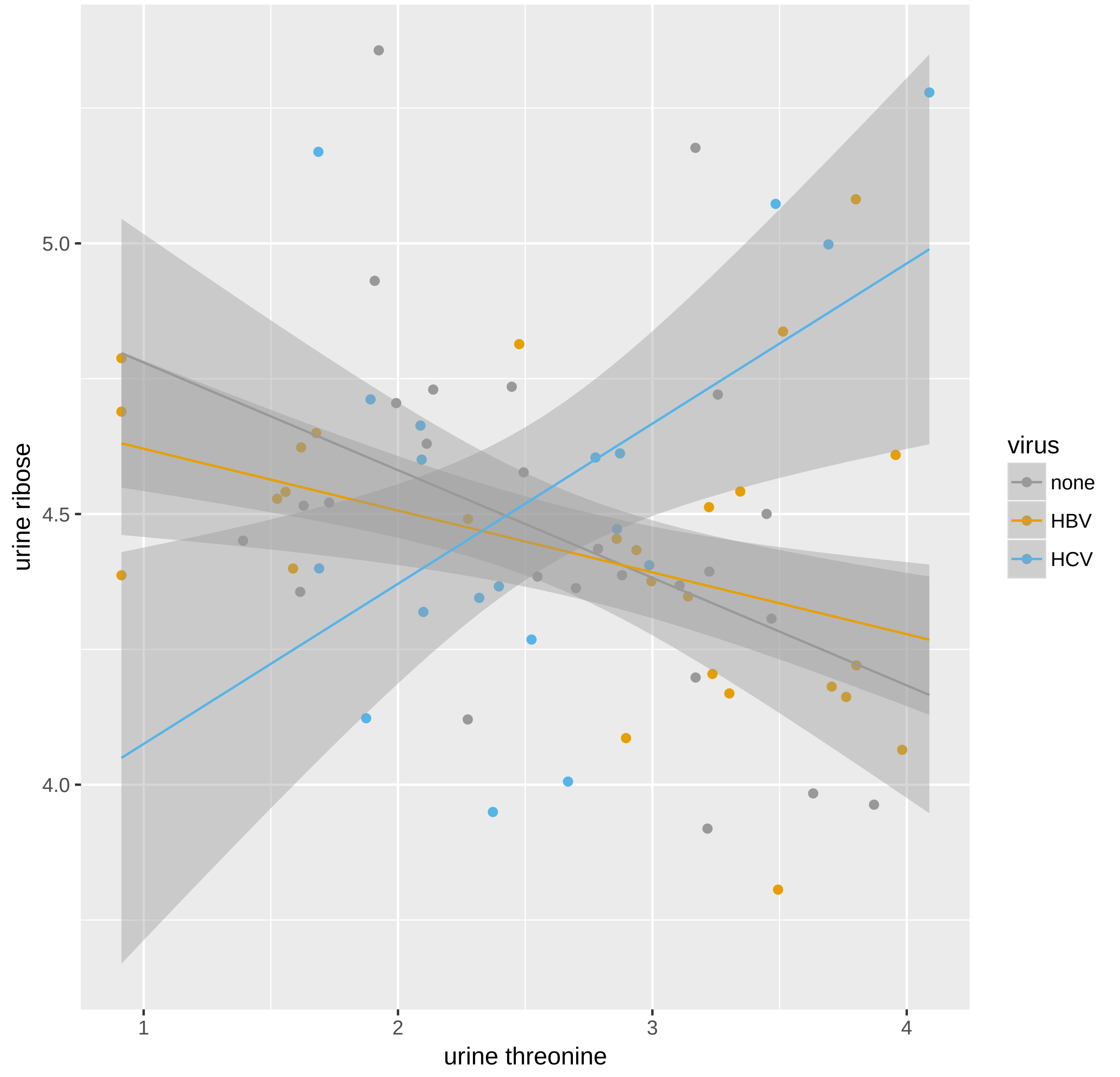

Figure S8. urine threonic acid and urine mucic acid

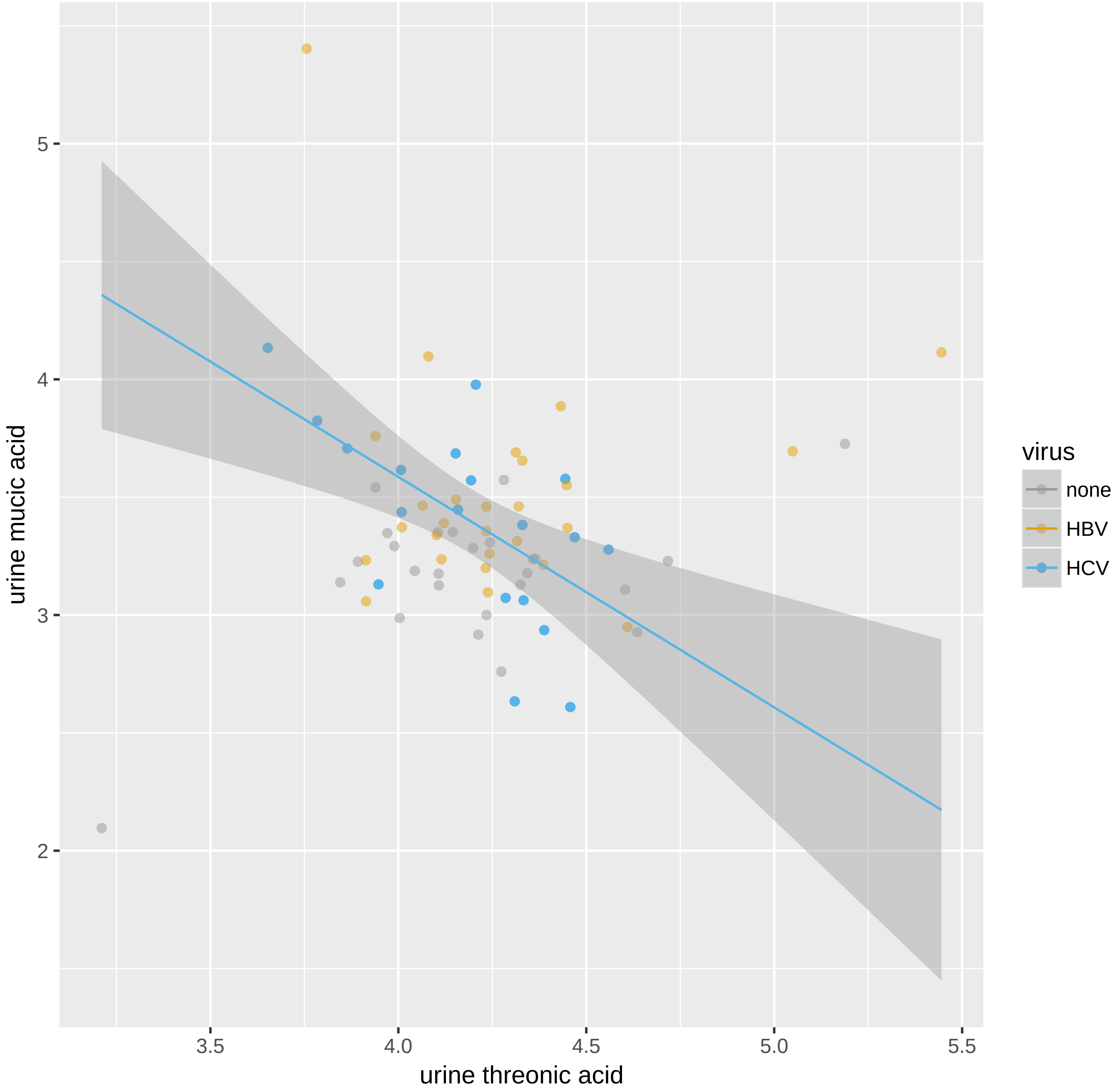

Figure S9. urine erythronic acid and urine citric acid

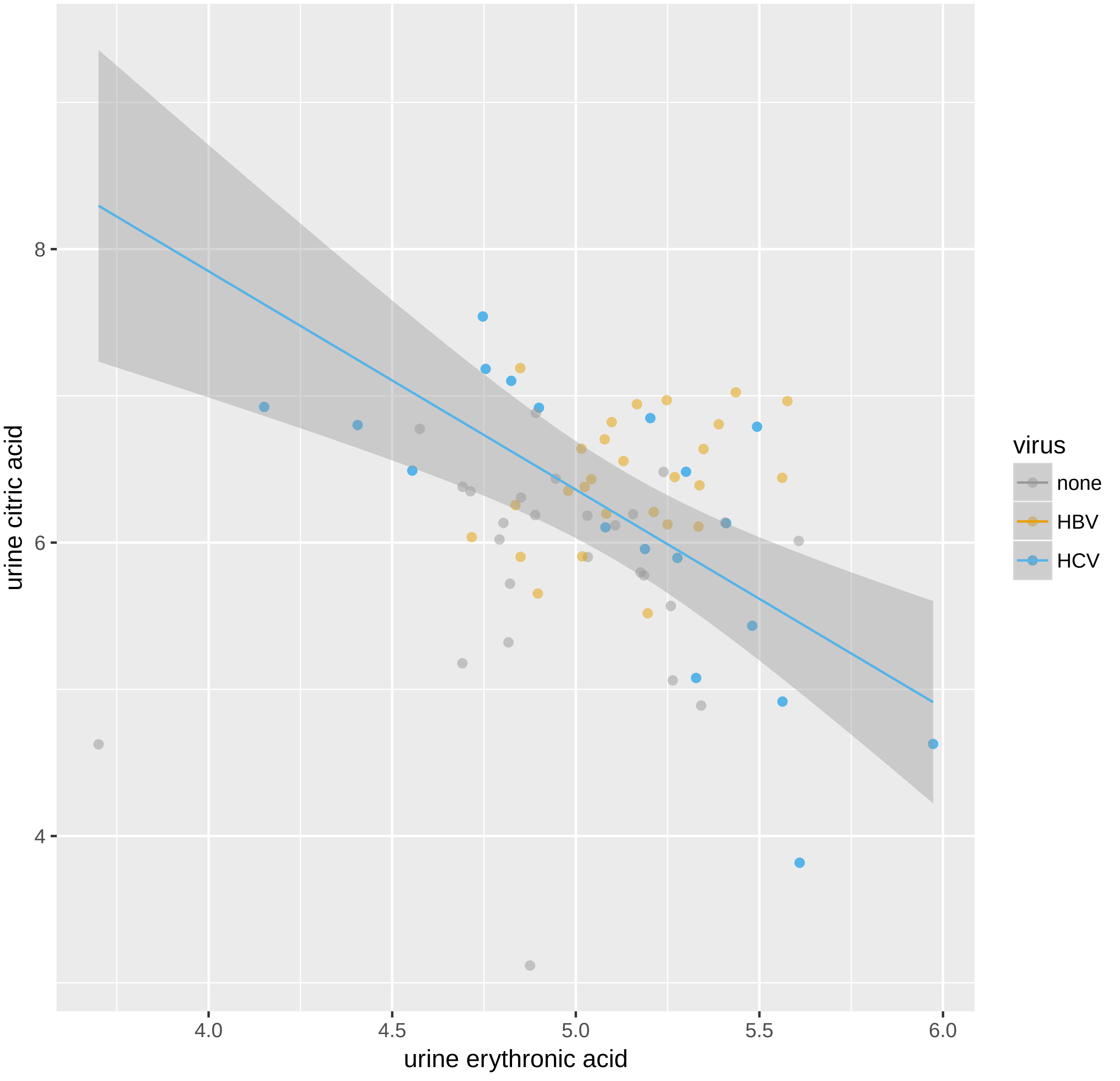

Figure S10. plasma myristic acid and plasma cholesterol

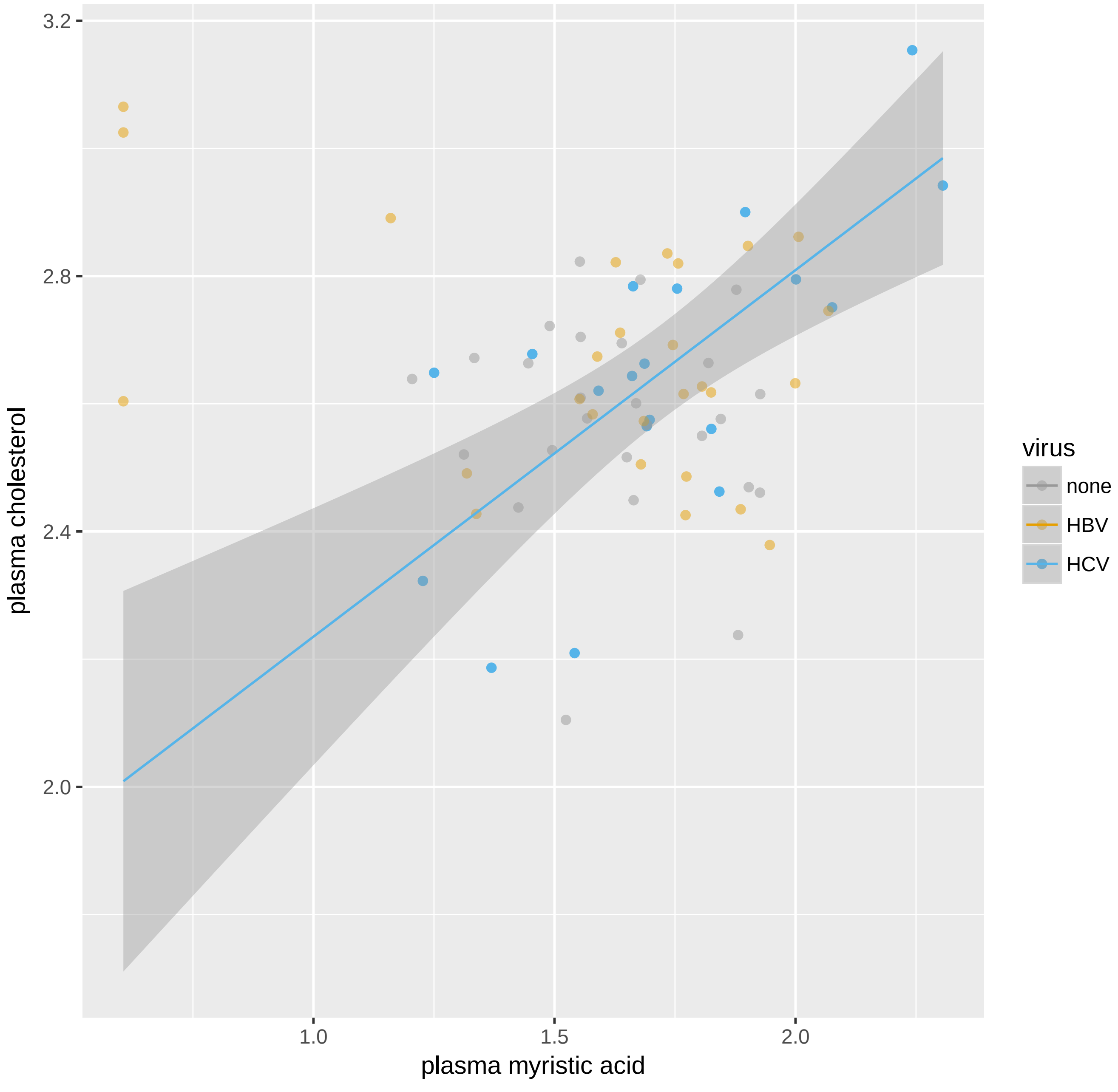

Figure S11. plasma valine and plasma serine

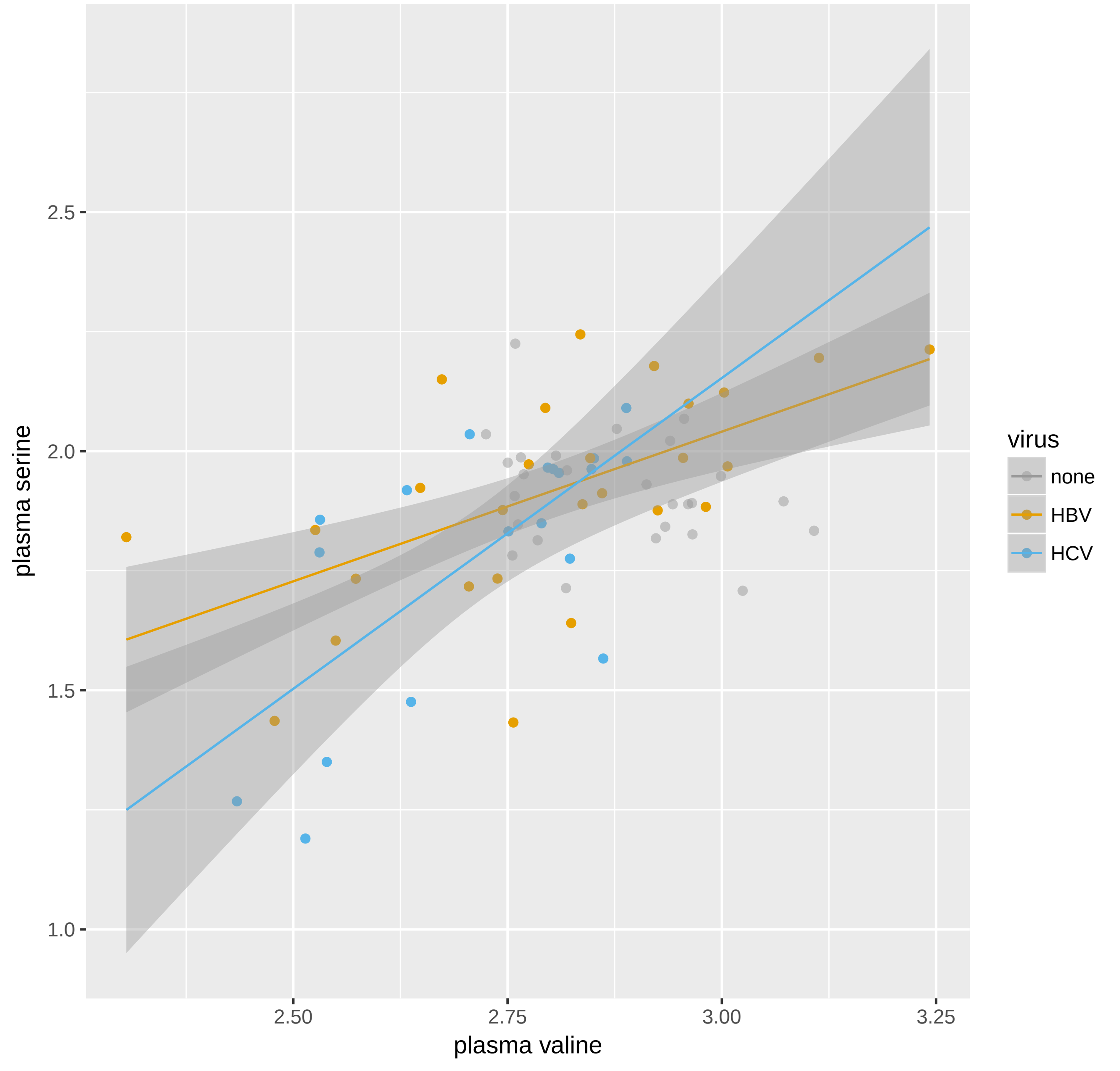

Figure S12. plasma isoleucine and plasma palmitoleic acid

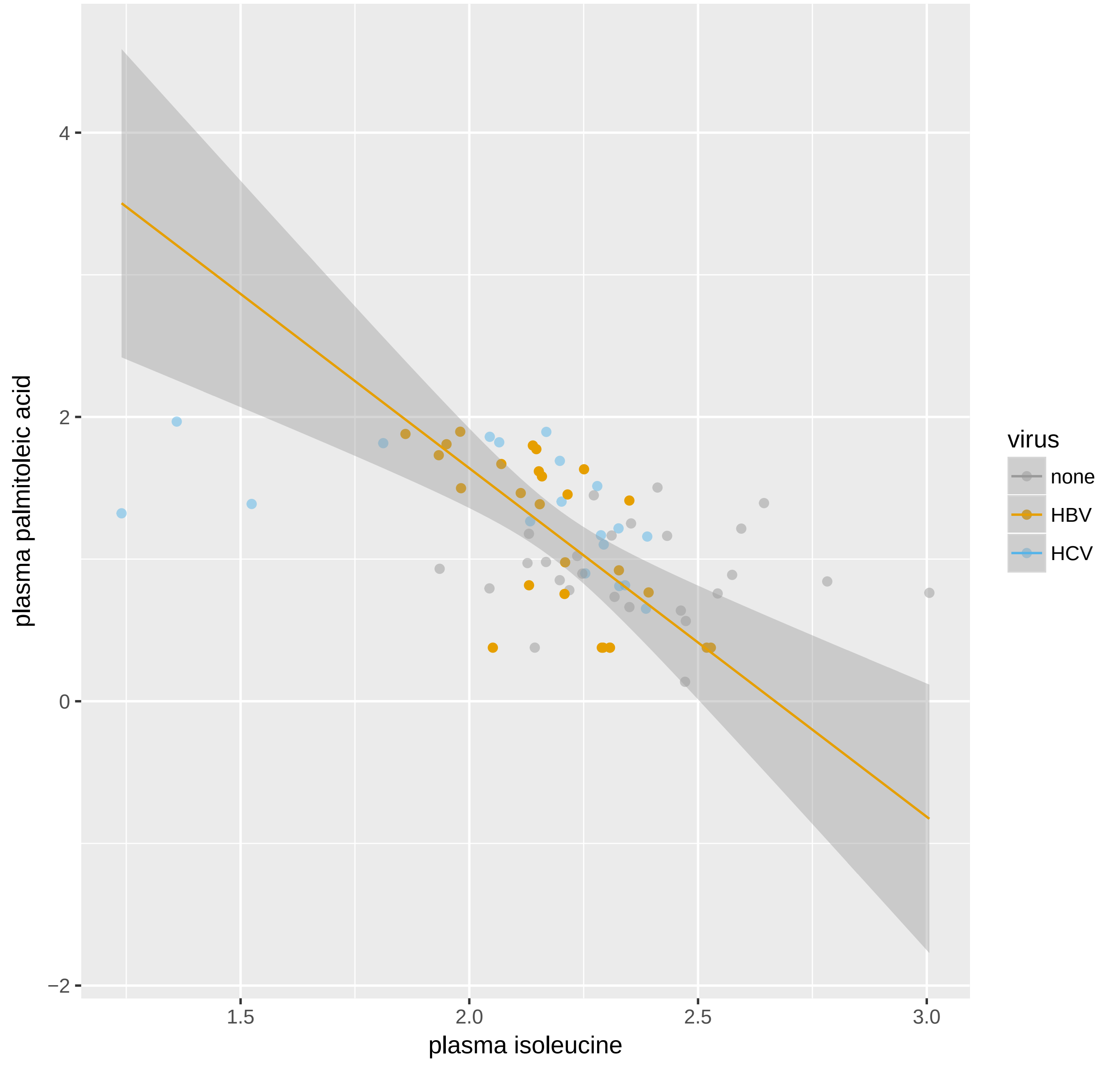

Figure S13. plasma isoleucine and plasma oleic acid

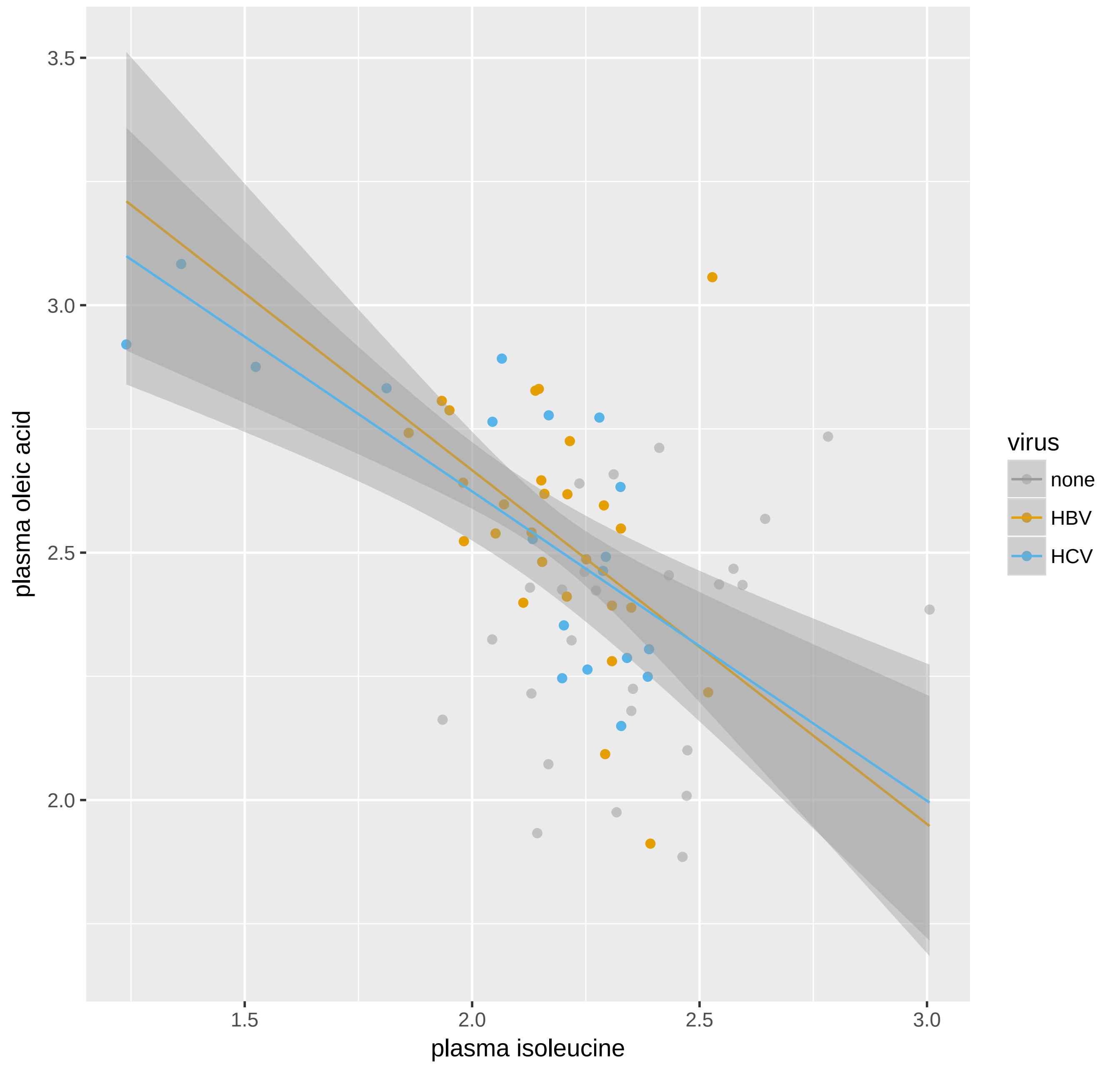

Figure S14. plasma leucine and plasma threonine

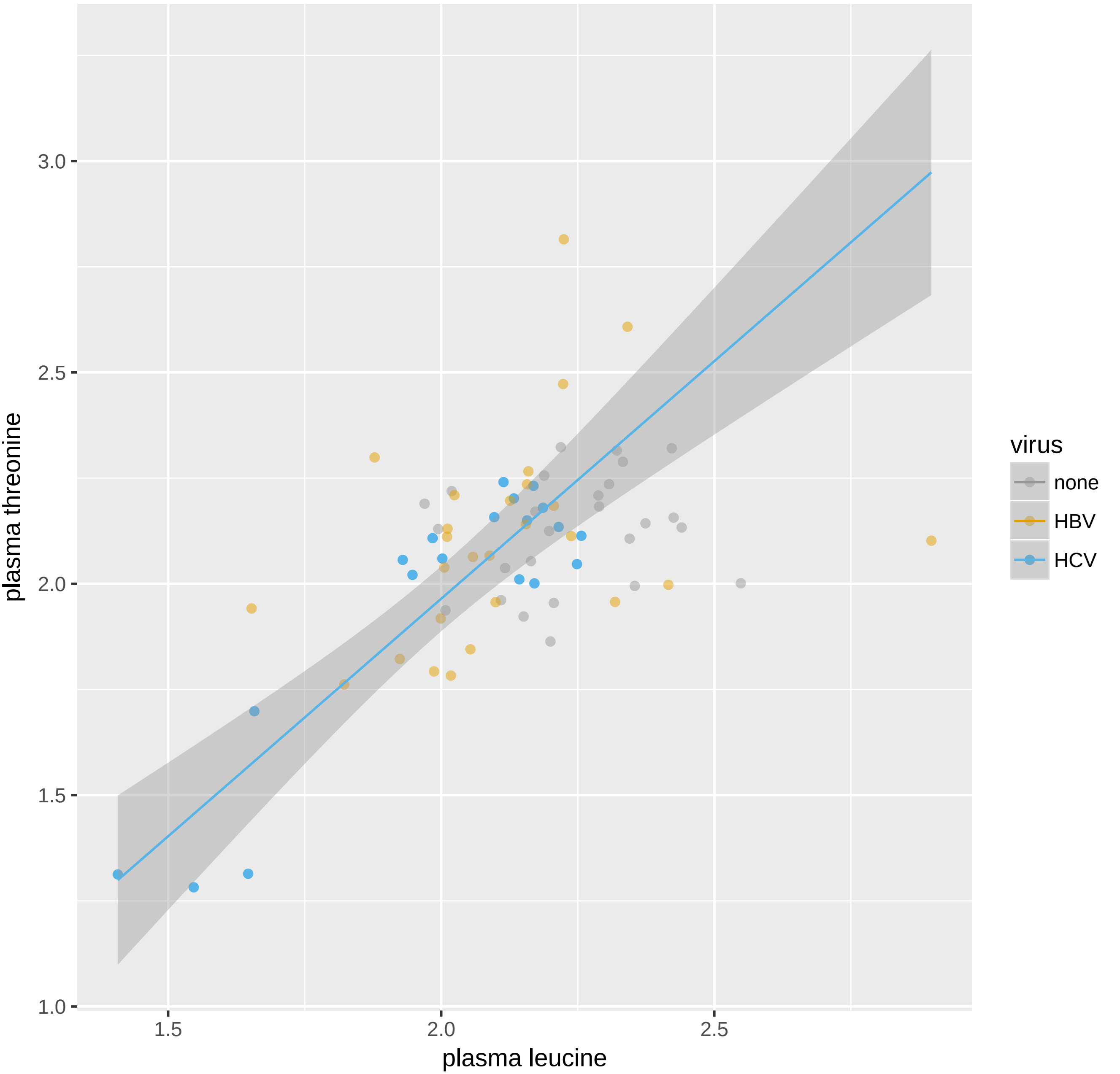

Figure S15. plasma isoleucine and plasma proline

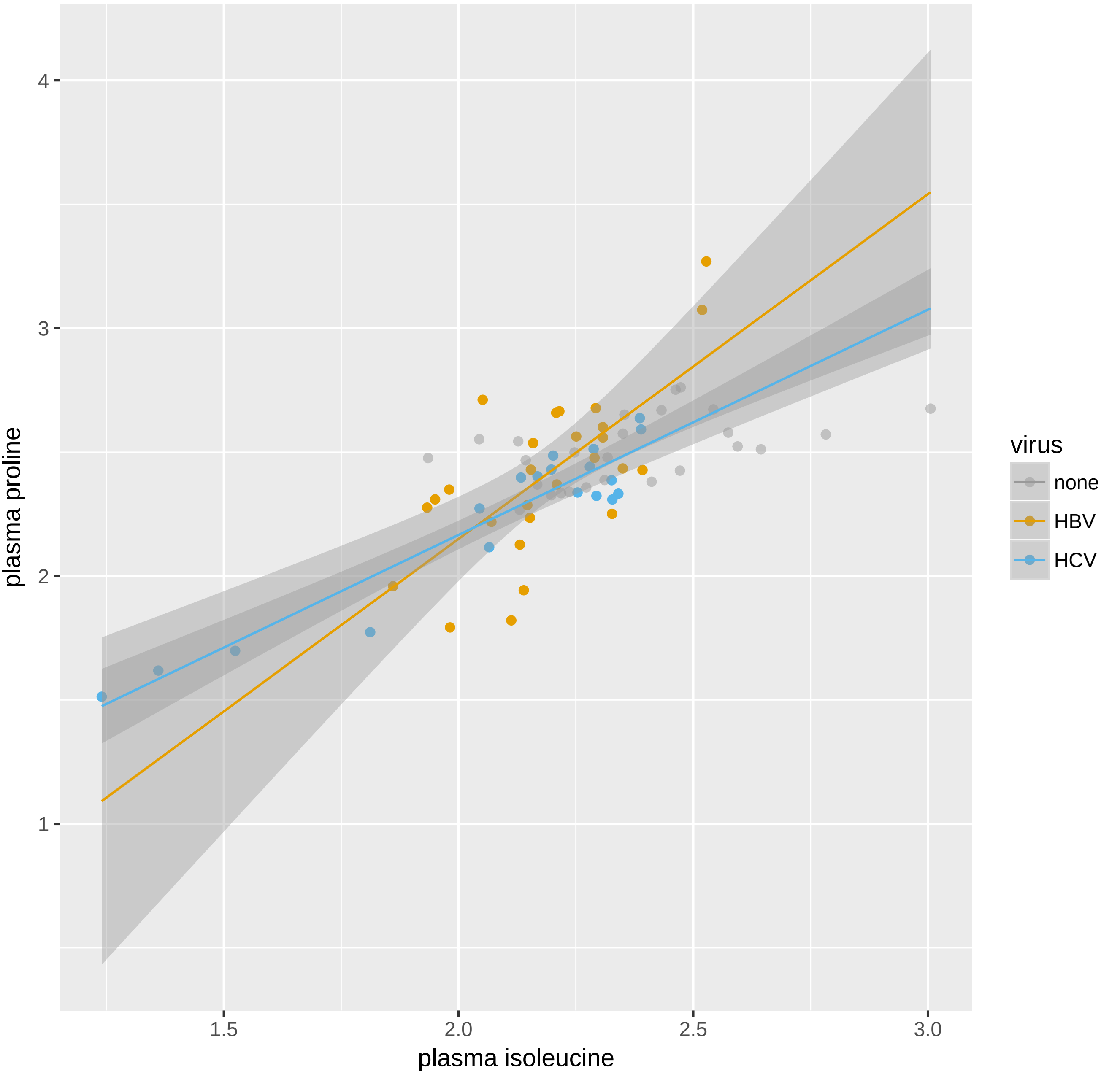

# Figure S16. urine xylitol and urine gluconolactone

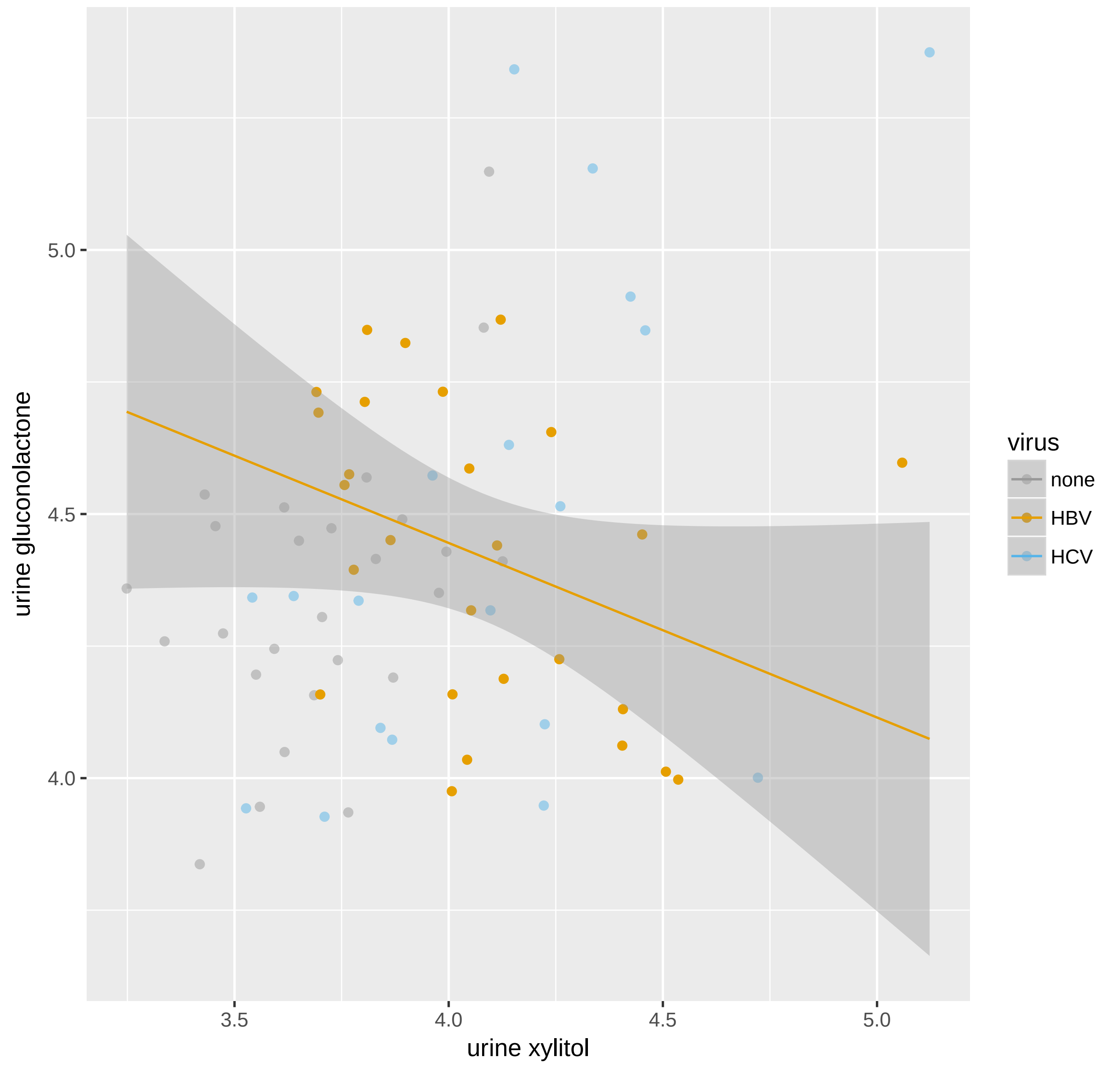

Figure S17. plasma valine and plasma glucose

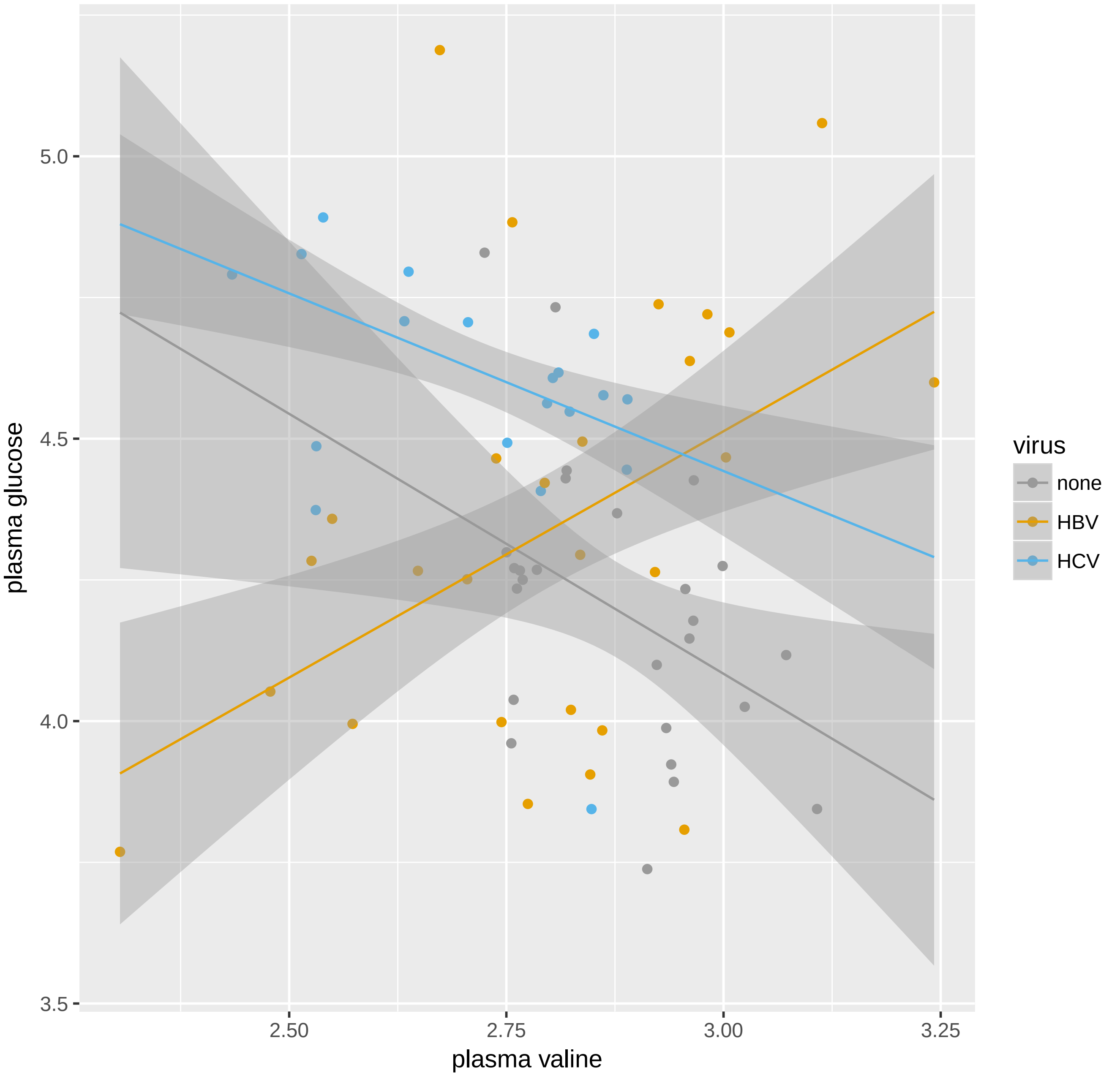

Figure S18. urine threonic acid and urine glucose

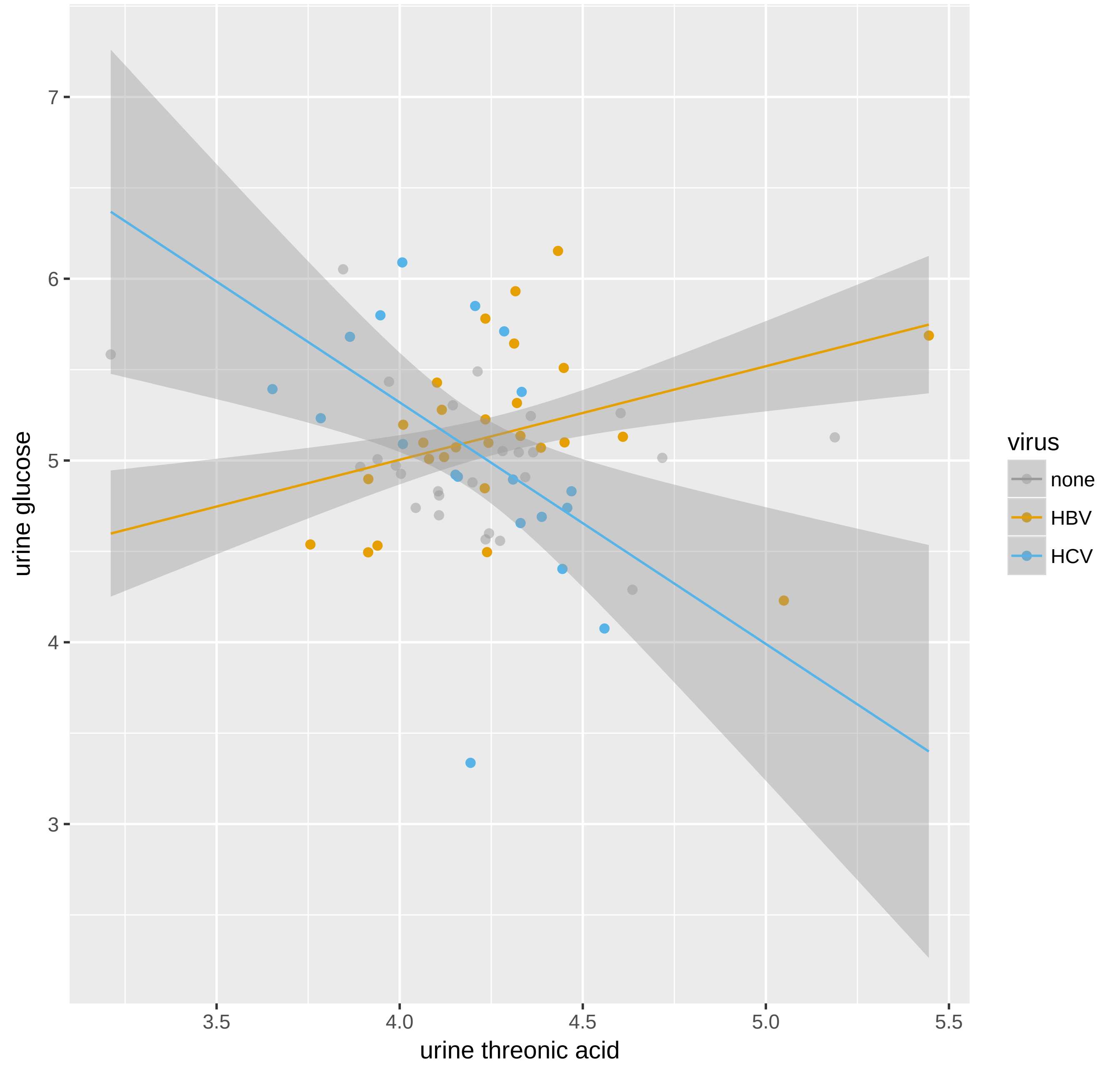

Figure S19. urine fructose and urine glucose

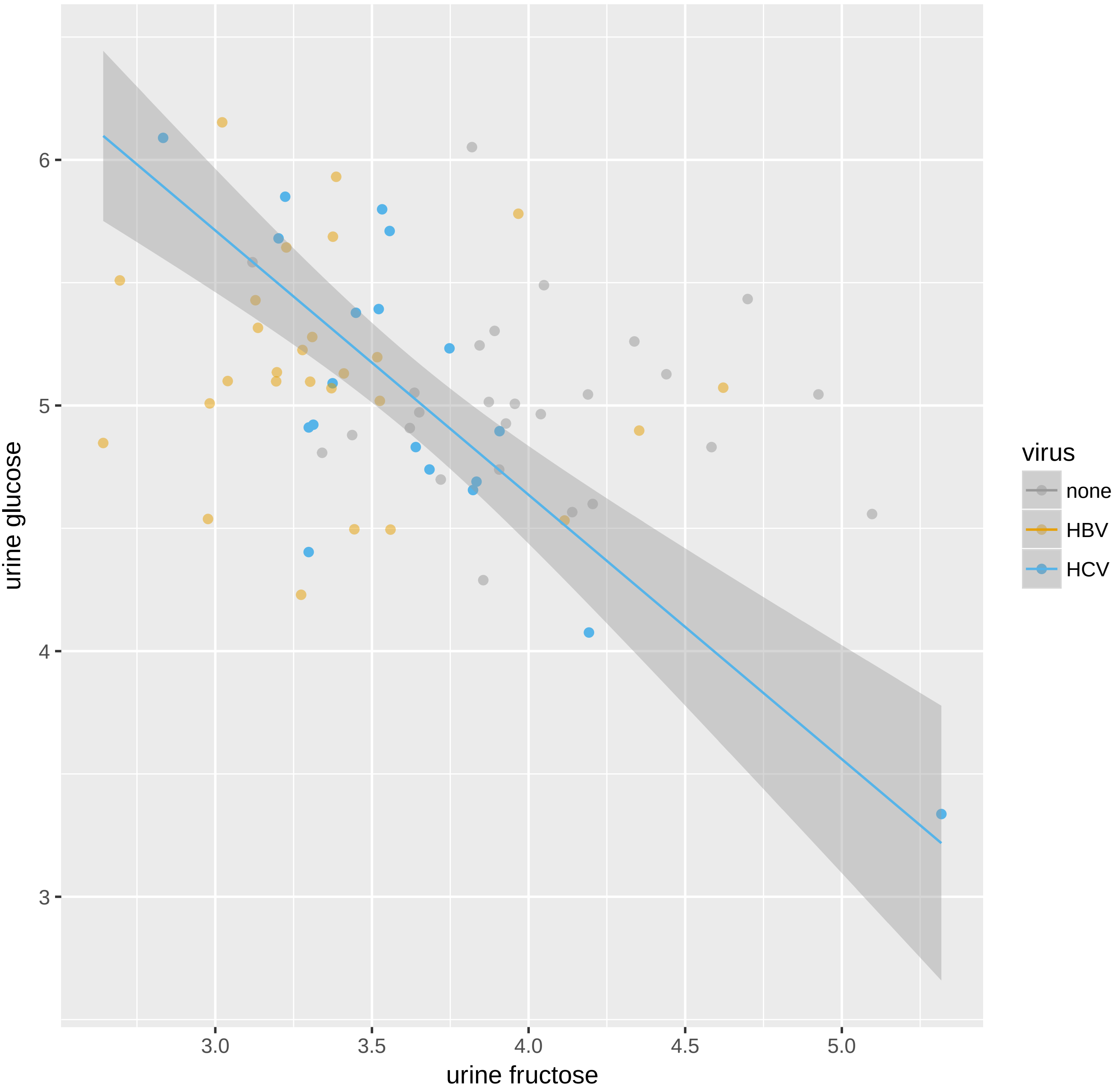

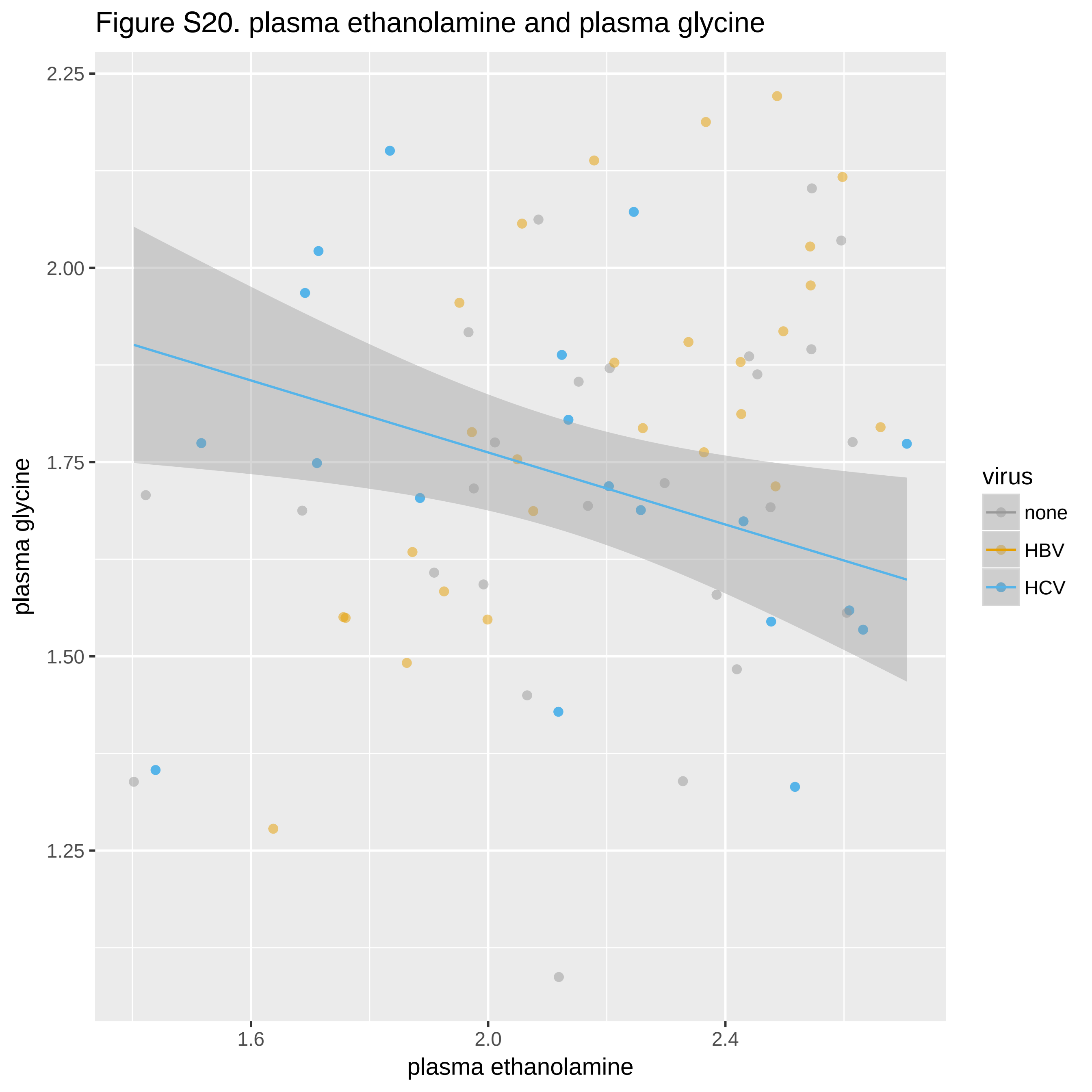

Figure S20. plasma ethanolamine and plasma glycine
plasma glycine
2.25
2.00
1.75
1.50
1.25
1.6
2.0
2.4
plasma ethanolamine
virus
none
HBV
HCV

Figure S21. plasma threonine and plasma mannose

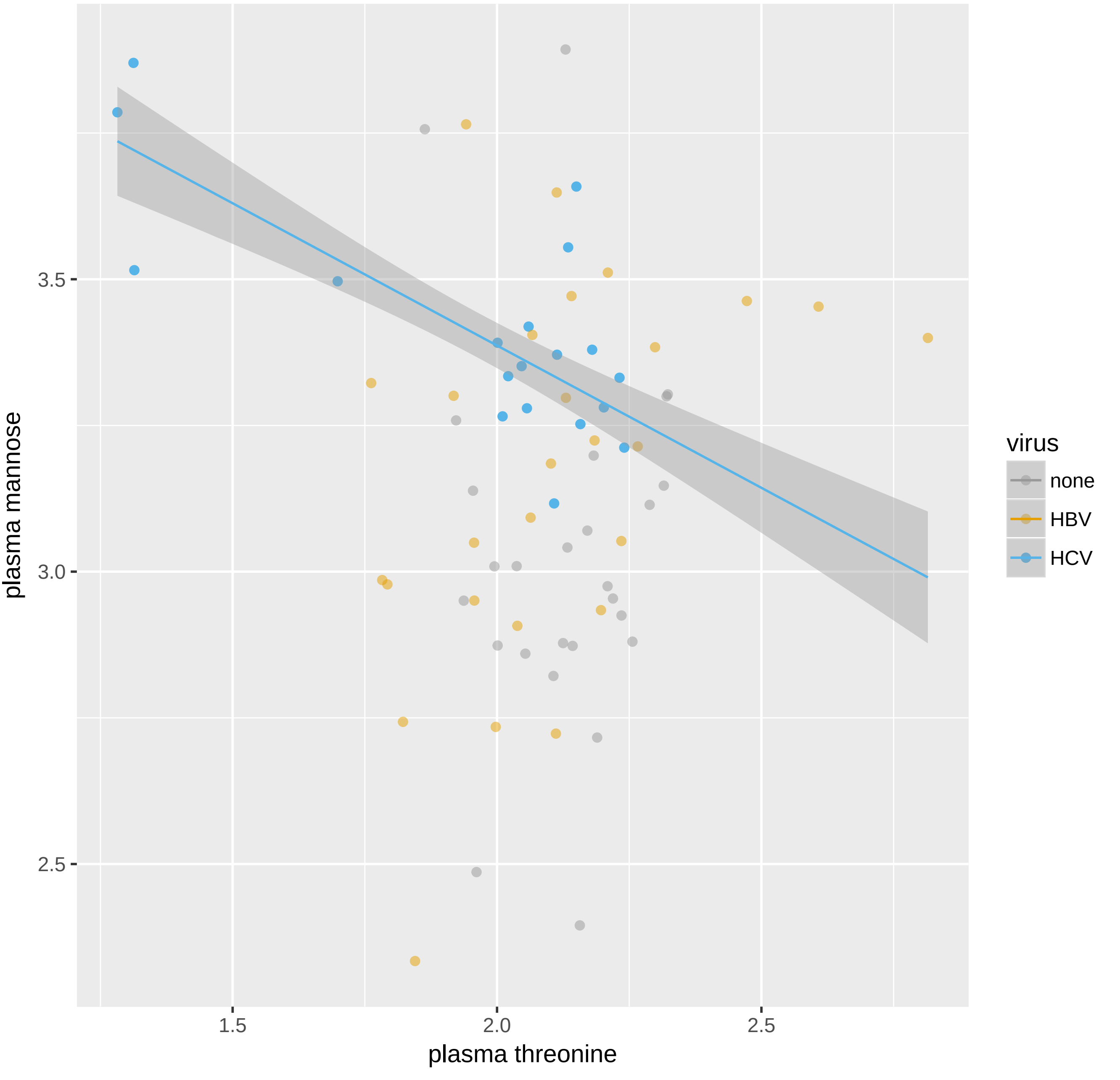

Figure S22. plasma serine and plasma myo−inositol

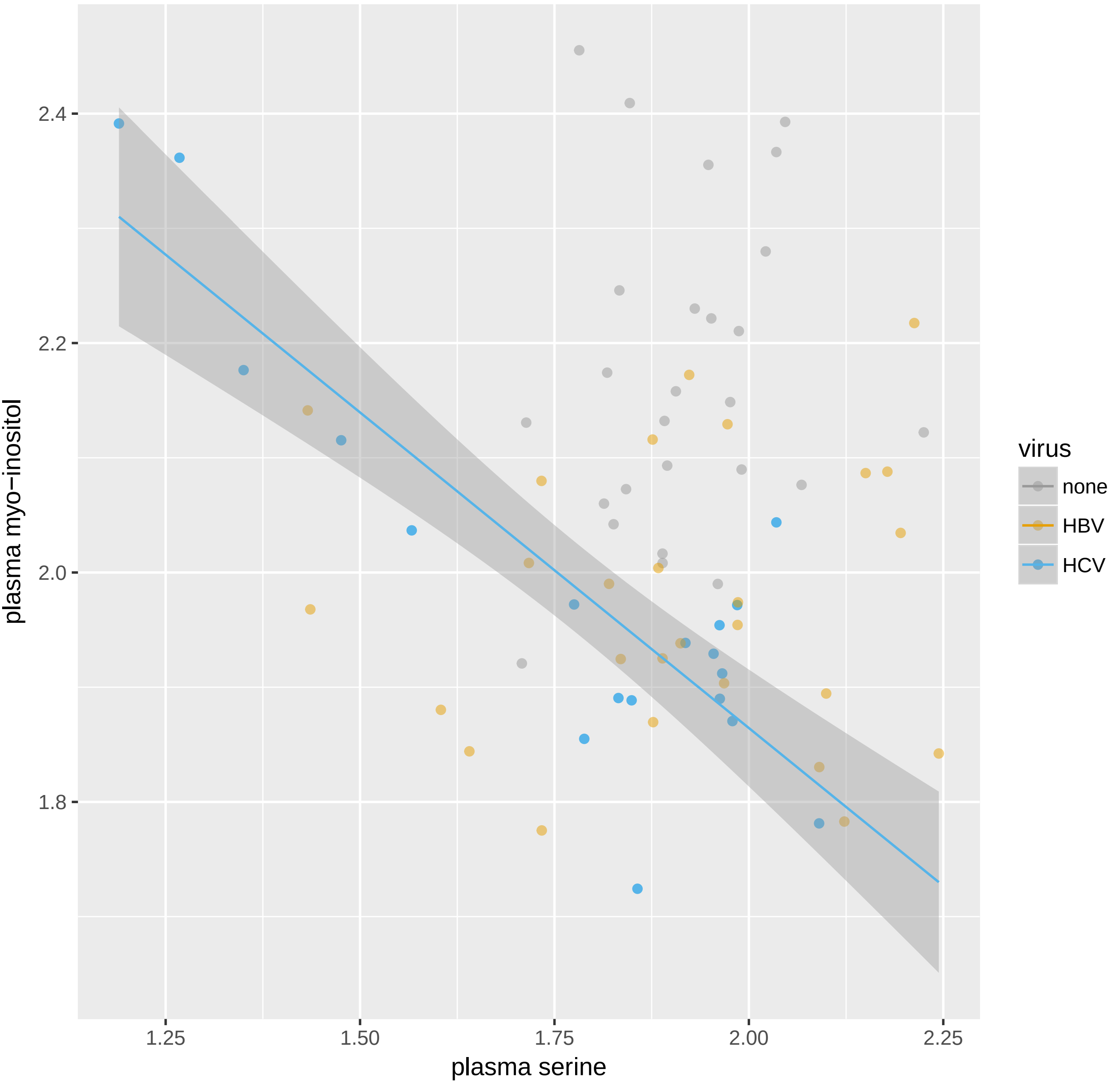

Figure S23. plasma oleamide and plasma cholesterol

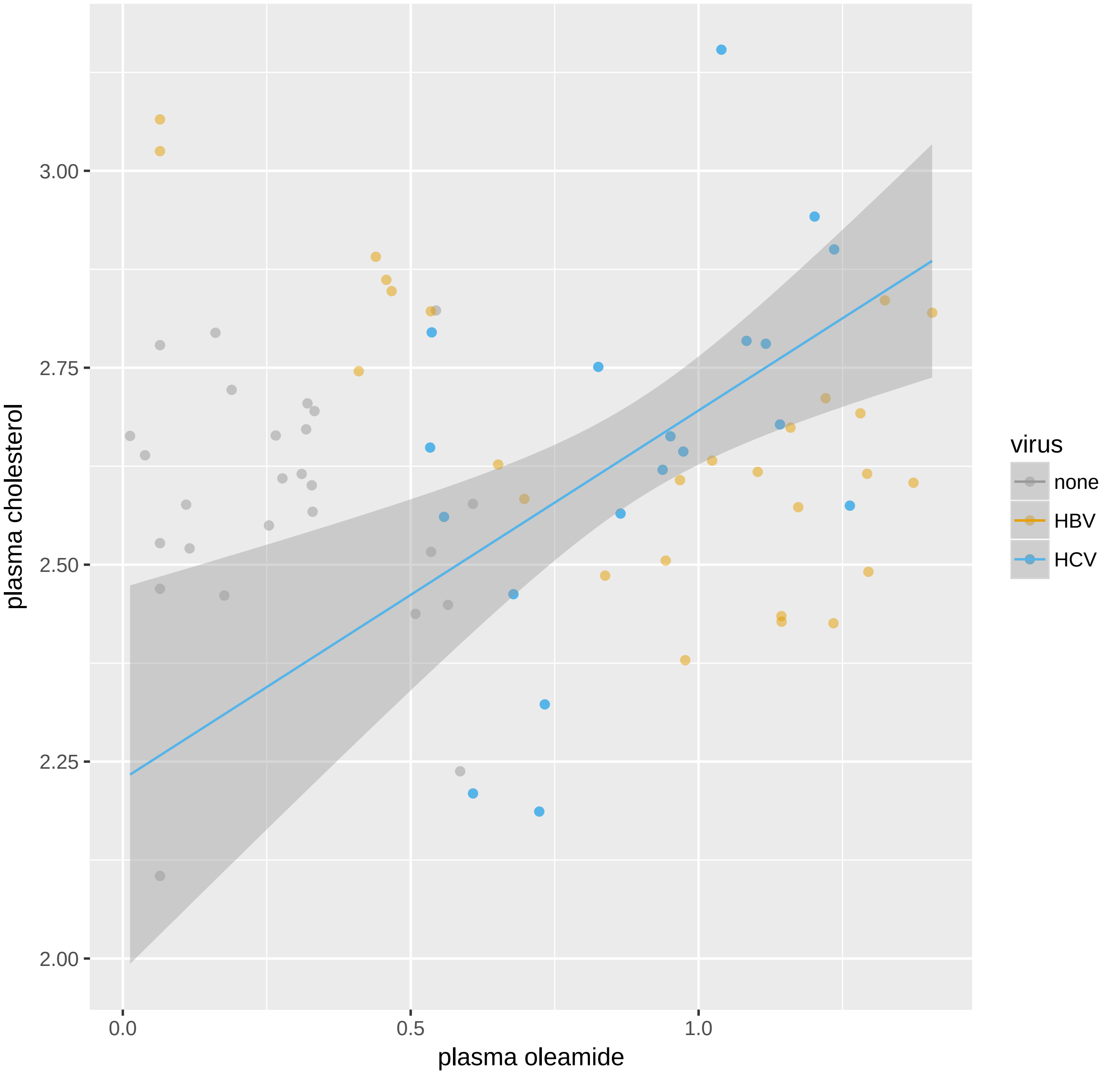

Figure S24. plasma leucine and plasma cholesterol

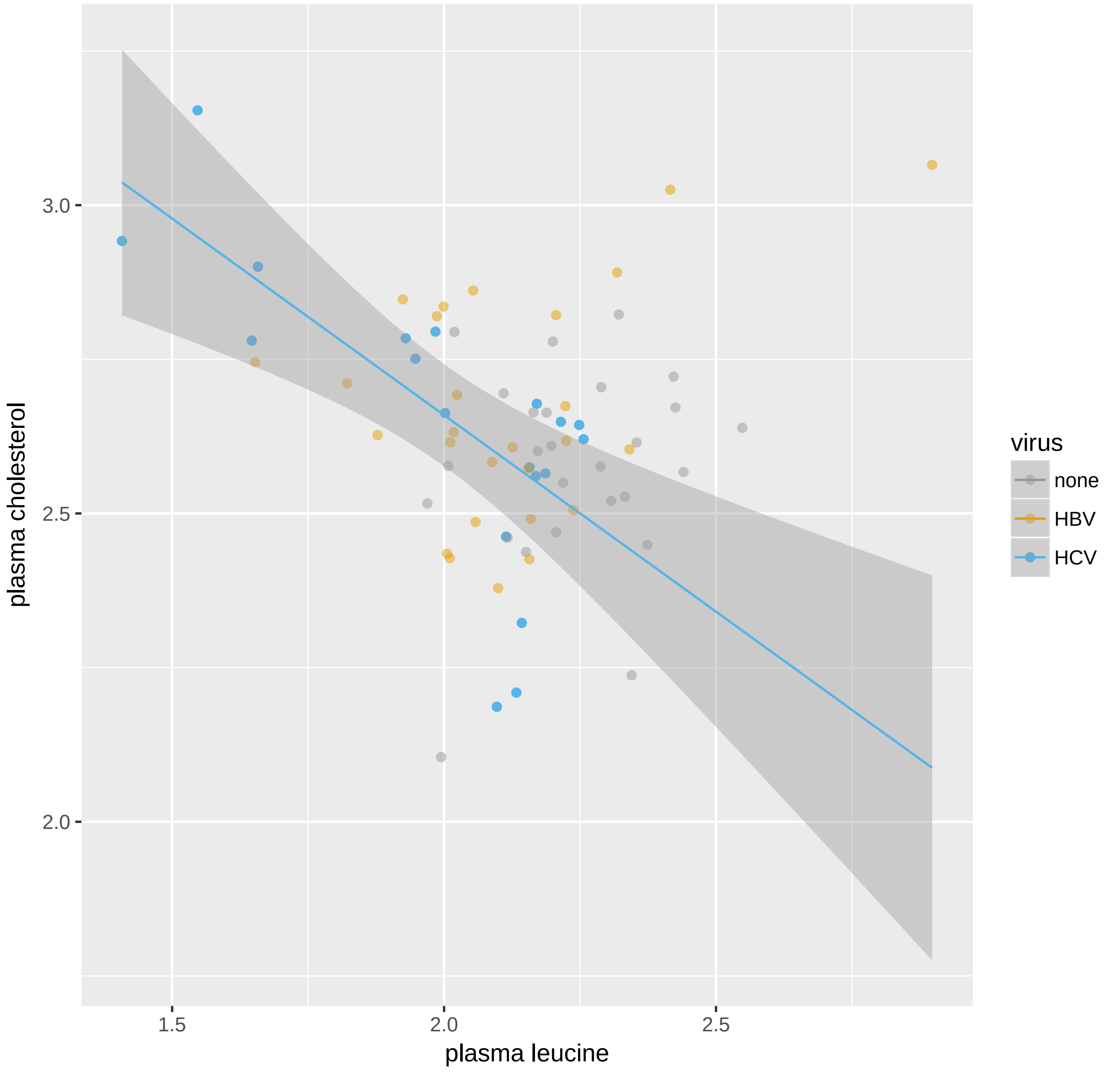

Supplement: Supplementary file 1 [file metabolites-07-00051-s001.pdf]
